# C‐Terminal Nucleobase Modification Amplifies Peptide‐Mediated Liposome Fusion

**DOI:** 10.1002/chem.202503665

**Published:** 2026-04-22

**Authors:** Laura Morbiato, Giacomo Bettin, Chiara Dalla Torre, Lorenzo Stella, Marta De Zotti

**Affiliations:** ^1^ Department of Chemical Sciences University of Padova Padova Italy; ^2^ Department of Chemical Science and Technology University of Rome Tor Vergata Rome Italy

**Keywords:** aggregation, cationic peptide, helical peptide, liposomes, nucleobase derivatization

## Abstract

The discovery of novel fusion peptides with enhanced properties is crucial for advancing our understanding of membrane fusion processes and developing more efficient tools for biotechnology and medicine. Currently, fusion peptides are mostly identified as portions of viral fusion proteins, and this limits their versatility. This study presents a significant step forward by demonstrating that end‐capping a cationic helical peptide with a nucleobase substantially enhances its ability to induce liposome fusion. We show that a helical trichogin analog, Oct‐K2569Tric‐Lol, containing four lysine residues, induces reversible aggregation of negatively charged liposomes in a concentration‐dependent manner. Remarkably, we found that the addition of a single thymine nucleobase at the C‐terminus of this peptide (Oct‐K2569Tric‐T) transforms its activity from reversible aggregation to irreversible membrane fusion. Furthermore, we observed that the presence of complementary nucleobases at both peptide termini promotes membrane fusion even at peptide‐to‐lipid ratios that do not cause membrane leakage. Our findings provide a new strategy for designing more potent fusion peptides, opening possibilities for improved applications in drug delivery, cell biology research, and nanotechnology.

## Graphical abstract

**Let's come together right now**. Cationic peptides are known to promote aggregation of negatively charged liposomes through electrostatic interaction. The process is reversible: once neutrality is achieved, liposomes disaggregate. Here, we demonstrate that end‐capping peptides with nucleobases turns the process into irreversible fusion.

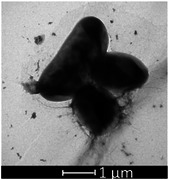

## 1 Introduction

Liposomes have an inherent ability to interact with cell membranes [1], but their capacity to actively fuse with the plasma membrane requires the presence of fusogenic agents [2]. This membrane fusion process can be exploited for direct delivery of encapsulated therapeutic cargo into the cell's cytoplasm, bypassing the endocytic pathway [3, 4]. Cationic peptides, especially amphipatic helical ones, are known to promote aggregation in negatively‐charged liposomes [5, 6] by inserting into the lipid bilayer, destabilizing it, and facilitating the fusion of two membranes through mechanisms that can involve the formation of membrane‐spanning α‐helices. Various studies have shown the importance of fusion peptides, portions of viral fusion proteins, in promoting enveloped virus entry, inserting into host cell membranes, and forcing the viral and host membranes to merge, allowing the virus to release its genetic material inside [7, 8]. The ability to promote aggregation of negatively‐charged liposomes plays an important, sometimes underestimated role in the mechanism of action of cationic antimicrobial peptides [9], and the identification of fusion peptides to be used as either antimicrobial agents or potential targets for therapy is a very active research topic [10, 11, 12]. Naturally‐occurring trichogin GA IV [13] is a useful model to study such peptide‐induced membrane aggregation. This short‐length membrane‐active, helical peptide (sequence reported in Table 1) is a member of the peptaibols family [14], possesses an amphipathic α‐helical structure, and can align either parallel or perpendicular to membranes depending on the membrane thickness and lipid composition.[15] It contains three residues of the tetrasubstituted α‐amino acid α‐aminoisobutyric acid (Aib) in its primary structure, which is a well‐known, strong, helix inducer [16]. Several cationic analogs of trichogin GA IV were reported in the literature, obtained by replacing one or more of the natural Gly residues with as many Lys. Those analogs were found to be membrane‐active and effective against several microbial pathogens,[17] including plant pathogens, with differences in antimicrobial activity depending on the number of Lys and the pathogen membrane.[18, 19] Aib‐containing, cationic peptides have already been found to promote liposome aggregation.[20] Efficient fusion between liposomes linked to two complementary coiled‐coil‐forming peptides was observed.[21] Besides, nucleobase‐functionalized lipids were found to promote fusion.[22, 23] Combining those results, here, we explore how end‐capping a helical peptide with one (or two) nucleobase(s) affects the peptide efficiency in promoting liposome aggregation/fusion. We previously reported the ability of peptides containing complementary nucleobases such as T‐K2569Tric‐A (Table 1) to self‐assemble forming filaments [24] and effectively mediate the electron transfer from a gold electrode to a photo‐excitable dye [25], behaving as a biomolecular wire. In this work, we first describe how one trichogin analog, Oct‐K2569Tric‐Lol (Table 1), containing four Lys residues, can induce reversible aggregation of negatively‐charged liposomes, with aggregation occurring when a certain peptide/lipid (P/L) ratio is reached, likely corresponding to neutralization of the negative membrane surface charge by the membrane‐bound cationic peptides. Disaggregation occurs by adding more peptide, thus inducing a positive charge on the liposomes. Then, we show how such reversible aggregation can be switched to fully irreversible membrane fusion simply by modifying the peptide sequence to include a single nucleobase—a thymine—at the C‐terminus (analog Oct‐K2569Tric‐T, Table 1). The presence of complementary nucleobases at both peptide termini also promotes membrane fusion, even at peptide‐to‐lipid ratios devoid of leakage activity.

**TABLE 1:** Sequences of the peptides synthesized and analyzed in this work.

Acronym	Sequence^a^
*Trichogin GA IV* ^b^	*Oct‐ Aib‐ Gly‐Leu‐Aib‐ Gly‐Gly‐ Leu‐Aib‐Gly‐Ile‐Lol*
Oct‐K2569Tric‐Lol	Oct‐Aib‐Lys‐Leu‐Aib‐Lys‐Lys‐Leu‐Aib‐Lys‐Ile‐Lol
T‐K2569Tric‐A	Thym‐Aib‐Lys‐Leu‐Aib‐Lys‐Lys‐Leu‐Aib‐Lys‐Ile‐Leu‐NH‐(CH_2_)_2_‐NH‐Ade
Oct‐K56Tric‐T	Oct‐Aib‐Gly‐Leu‐Aib‐Lys‐Lys‐Leu‐Aib‐Gly‐Ile‐Leu‐NH‐(CH_2_)_2_‐NH‐Thym
Oct‐K259Tric‐T	Oct‐Aib‐Lys‐Leu‐Aib‐Lys‐Gly‐Leu‐Aib‐Lys‐Ile‐Leu‐NH‐(CH_2_)_2_‐NH‐Thym
Oct‐K2569Tric‐T	Oct‐Aib‐Lys‐Leu‐Aib‐Lys‐Lys‐Leu‐Aib‐Lys‐Ile‐Leu‐NH‐(CH_2_)_2_‐NH‐Thym
Ac‐K2569Tric‐T	Ac‐Aib‐Lys‐Leu‐Aib‐Lys‐Lys‐Leu‐Aib‐Lys‐Ile‐Leu‐NH‐(CH_2_)_2_‐NH‐Thym

^a^ Thym, thymine‐1‐acetyl; Ade, adenine‐9‐acetyl; Aib, α‐aminoisobutyric acid; Ac, acetyl; Oct, *n‐*Octanoyl; Lol, 1,2‐aminoalcohol Leucinol. All amino acid residues are L.

^b^ The sequence of the naturally occurring peptaibol trichogin GA IV is reported for comparison.

## 2 Results and Discussion

### 2.1 Synthesis

Aiming at investigating the effect of the presence of nucleobases at the N‐ and/or C‐terminus of a cationic, helical peptide on its ability to promote aggregation of model liposomes we synthesized by a combination of manual solid‐phase peptide synthesis (SPPS) and synthesis in solution four peptides containing an increasing number of Lys residues (Table 1). Each peptide sequence was grown on a 2‐chlorotrytil resin preloaded with an ethylendiamine linker and then cleaved, leaving all side‐chains protected (details in the Experimental). C‐terminal capping with either thymine‐1‐acetyl (Thym) or adenine‐9‐acetyl (Ade) was performed in solution.

The main peptide sequence, called Oct‐K2569Tric‐Lol, is an analog of the naturally occurring peptide trichogin GA IV, in which the four Gly residues were replaced by as many Lys (Table 1). Oct‐K2569Tric‐Lol has been found to effectively interact with membranes, causing their leakage [17], and to be active against human [25] and plant pathogens [17, 18]. Its ability to induce aggregation of model membranes was never explored before. T‐K2569Tric‐A was synthesized as previously described [24]. The removal of the naturally occurring *N*‐terminal Octanoyl group from trichogin is known to cause a loss of its ability to interact with model membranes. Therefore, we decided to place the nucleobase at the C‐terminus for all peptide analogs synthesized in this work (Table 1). To this aim, we activated in solution either Thym or Ade, synthesized as previously described [26], with *N*‐(3‐Dimethylaminopropyl)‐*N*'‐ethylcarbodiimide hydrochloride (EDC) and 1‐Hydroxybenzotriazole (HOBt) and added the peptide, sinthesized by manual SPPS and cleaved from the resin under mild conditions [hexafluoroisopropanol (HFIP) 30% in CH_2_Cl_2_] to preserve the side‐chain protective groups (crude yields between 60 and 85%; crude purity 50%–60%). To investigate if a less efficient membrane interaction might result in a reduced leakage from liposomes during their fusion, we also synthesized the acetylated analog Ac‐K2569Tric‐T (Table 1). We purified each peptide at that stage to ≥ 94% purity, by medium‐pressure reverse‐phase chromatography (Biotage Isolera Prime instrument), exploiting the high hydrophobicity of the fully protected trichogin analogs. After deprotection with HCl 3 M in methanol, the peptides were obtained by precipitation from ethyl ether. No further purification steps were needed. Characterization by High‐Resolution Electron Spray Ionization Mass Spectroscopy (HR‐ESIMS), ^1^H and ^13^C NMR of the newly synthesized peptides is reported in the Supporting Information.

### 2.2 Circular Dichroism (CD)

**FIGURE 1:**
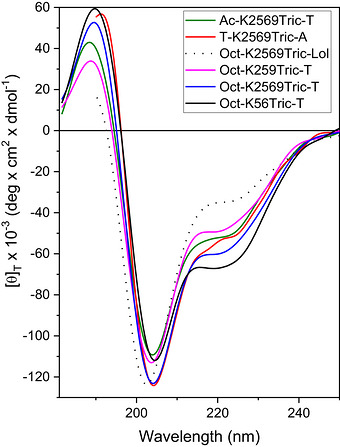
Dichroic profiles ([θ]_T_, total ellipticity) recorded in distilled water (pH 6) for the newly synthesized trichogin analogs, bearing a Thymine‐acetyl moiety at the C‐terminus, and for the two reference peptides (reported for comparison). Peptide concentration: 10^−4^ M.

Both the naturally occurring peptide trichogin GA IV and its Lys‐containing analogs are known to adopt a right‐handed, mixed 3_10_‐/α‐helical conformation regardless of the experimental conditions [25], and their well‐developed helix is known to promote effective interaction with the biological membranes. To assess the effect of the presence of both a different number of Lys residues and a single nucleobase moiety at the C‐terminus on the conformational preferences of the reference sequences, we performed a CD study in water (Figure 1), a solvent that does not support or stabilize any conformations, allowing for an evaluation of the conformation stability, as well. For comparison, we recorded also the CD profiles of the two reference peptides under the same experimental conditions. The spectra of all analogs feature a positive maximum centered at about 195 nm, and two negative maxima at about 205 and 222 nm, namely the canonical positions for a right‐handed helical 3D‐structure [27]. The CD profiles suggest the presence of a mixed 3_10_‐/α‐helical conformation for all analogs, that is, the 3D‐structure adopted by the native peptide trichogin GA IV and reported for other Lys‐containing trichogin analogs [25].

Even if trichogin analogs are short peptides (11‐residue long), their helix appears well defined and stable in water, an environment that does not promote any type of 3D structure. Table 2 reports the *R* ratio between the total ellipticities recorded at the two negative maxima, centered at 220 and 205–208 nm. A *R* value below 0.3 and a blue shift of the negative maximum at about 208 nm to ca. 205 nm are both indicative of the presence of a 3_10_‐helix.

**TABLE 2:** Ellipticity [θ] (deg cm^2^ dmol^−1^) registered at the two negative maxima, and the ratio *R*, for all peptides.

Peptide	[θ]_208_	[θ]_220_	*R* [θ]_220_/[θ]_208_
Oct‐K2569Tric‐Lol	−123.6630	−35.1185	0.28
Oct‐K2569Tric‐T	−123.1808	−60.1270	0.49
Ac‐K2569Tric‐T	−109.283	−52.2074	0.48
T‐K2569Tric‐A	−124.1600	−54.2491	0.44
Oct‐K259Tric‐T	−113.0560	−49.2350	0.44
Oct‐K56Tric‐T	−111.9795	−67.0360	0.60

Both the dichroic profiles (Figure 1) and data reported in Table 3 give clear indications that the substitution of the C‐terminal 1,2‐aminoalcohol Lol with a Leu‐Thymine (or Adenine) does affect the conformational preferences of the sequence in water. While the reference compound Oct‐K2569Tric‐Lol has an R value of 0.28, diagnostic of an almost fully 3_10_‐helical structure, the addition of Thym at the C‐terminus shifts the R value toward 0.5, or even 0.6, indicating a higher percentage of α‐helix. This behavior has already been observed for trichogin analogs bearing a C‐terminal primary amide, namely a ‐Leu‐NH_2_ [17]. We consider this result as originating from the additional H‐bond pseudocycle involving the 1,2‐diaminoethane linker. Oct‐K2569Tric‐Lol was modelled in a 3_10_‐helical conformation [17], finding that the four basic amino acid residues are all lying on the hydrophilic face of the amphipathic helix, with the side chains eclipsed. We suspect that the presence of the additional H‐bond may allow the structure to switch to a mixed α‐/3_10_‐helical conformation, with staggered, noneclipsed side chains. Modifications to the N‐terminal capping moiety do not seem to affect the overall helical conformation of the peptides. We extended our CD analysis to gain information on the stability of the peptide 3D‐structure in liposomes and at different peptide concentrations. To this aim, we acquired the CD profiles of Oct‐K2569Tric‐Lol, Oct‐K2569Tric‐T, and T‐K2569Tric‐A in water at 10^−3^, 10^−4^, and 10^−5^ M concentration, and in the presence of liposomes made of palmitoyl oleoyl phosphatidylethanolamine (POPE) and phosphatidylglycerol (POPG) 7:3. The CD profiles acquired for each peptide at different peptide concentrations were almost superimposable (see Figure S41, Supporting Information). The CD analysis in the presence of liposomes for all three peptides highlighted the presence of a fully‐developed α‐helix, with the negative maxima centered at about 208 and 220 nm (see Figure S42, Supporting Information). This observation is in line with the literature that reports that Lys‐containing trichogin analogs tend to adopt an α‐helical conformation in the presence of micelles or liposomes.[17]

**TABLE 3:** Average values of the hydrodynamic diameter (z‐averaged size) and standard deviation obtained by DLS analysis (scattering angle: 173°, measures done in triplicate) of SUVs PEPG treated with each peptide at the three peptide/lipid ratios: 1:15, 1:6, and 1:3.

Acronym	Hydrodynamic diameter nm peptide/lipid ratio: 1:15	Hydrodynamic diameter nm peptide/lipid ratio: 1:6	Hydrodynamic diameter nm peptide/lipid ratio: 1:3
Pristine liposomes	112.5±0.6		
Oct‐K2569Tric‐Lol	195.6±2.7	5822±1880	106.9±0.06^a^
Oct‐K2569Tric‐T	327±17	7484±4553^b^	18750±16570^b^
Ac‐K2569Tric‐T	316.4±8.7	—	13200±5247^b^
Oct‐K56Tric‐T	78.1±1.5	107.8±2.1	28920±15460^b^
Oct‐K259Tric‐T	162.4±7.8	147.3±6.6	7830±3466^c^
T‐K2569Tric‐A	157.3±4.1	3191±173	948±25^d^

^a^ No high‐dimension particles were detected at P/L 1:2, namely the highest peptide/lipid ratio tested, either (hydrodynamic diameter at P/L 1:2 92.7 ± 1.4 nm, see Supporting Information).

^b^z‐averaged diameter calculated by the instrument over the three repetitions, even when one or two of the measurements detected diameters out of range (> 10'000nm), see Supporting Information.

^c^At a peptide‐to‐lipid ratio of 1:3, the DLS detects a majority of particles with huge dimensions, about 10^4^ nm;

^d^This value is probably underestimated, since at a peptide/lipid ratio of 1:2 the instrument detected a z‐average diameter of 10530±8604 nm.

### 2.3 Leakage Experiments

**FIGURE 2:**
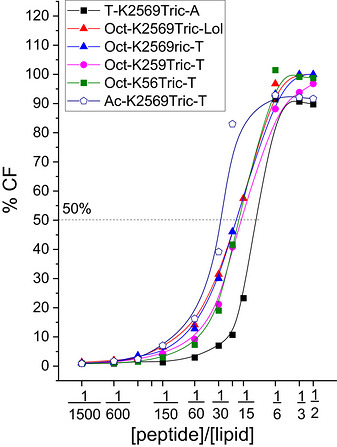
CF leakage from SUVs (lipid composition PE/PG 7:3; lipid concentration, 60 µM) as a function of P/L ratios, induced by increasing peptide concentration.

The ability of peptides to induce leakage of liposome contents entrapped in liposomes was checked by monitoring the change in the fluorescence intensity of carboxyfluorescein (CF), which had been encapsulated in liposomes at high self‐quenching concentrations. We tested the ability of the peptides to modify the permeability of small, negatively charged unilamellar vesicles (SUVs) composed of a mixture of palmitoyl oleoyl phosphatidylethanolamine (POPE) and phosphatidylglycerol (POPG) 7:3, thereby called PEPG SUVs. Results are reported in Figure 2. All peptides were able to cause CF release via a cooperative mechanism, as suggested by the sigmoid trend, but they reach 50% of the total release at different P/L ratios. The N‐Octanoylated analogs were able to cause 50% release of the dye at P/L ratios of about 1/30. Cationic peptides can promote aggregation of negatively charged liposomes, we were not surprised when we observed turbidity in the solutions of the leakage experiments, especially at high peptide‐to‐lipid (P/L) ratios (i.e., 1:6). T‐K2569Tric‐A showed the highest P/L ratio for 50% CF leakage, namely 1:15. In that experiment, we observed turbidity at lower P/L ratios than for K2569Tric, which we interpreted as a visible sign of early liposome aggregation. Of course, those experiments are meant only to evaluate the ability of the peptides to modulate the membrane permeability, not to show fusion or aggregation. To investigate the ability of peptides to induce vesicle fusion, we performed Dynamic Light Scattering, Transmission Electron Microscopy, and Isothermal titration calorimetry (see below) on the same solutions. Because of expected problems with fluorescence intensity, due to interference from the aromatic nucleobases [28], proving or quantifying lipid/content mixing was not attempted.

Literature reports the important role played by the N‐terminal octanoyl moiety on trichogin's ability to cause membrane leakage.[29] In particular, C. Toniolo et al. [30] reported permeability measurements performed on analogs of the naturally occurring peptide trichogin GA IV (without positively charged residues) carrying at the N‐terminus an acyl chain of variable length, and revealed that at least six carbon atoms in the N*R*‐blocking fatty acyl moiety are required for the onset of significant membrane‐modifying properties. In that work, N‐acetylated trichogin was found completely unable to cause membrane permeabilization. Indeed, in our experiments, the presence of either an Acetyl or a Thymine‐acetyl at the N‐terminus limited the maximum leakage to about 90%. The membrane activity in this case was not totally lost: the enhanced amphipathicity of the peptide due to the presence of the Lys residues probably counteracted the effect of the N‐terminal capping moiety. This observation is of interest for future applications in drug delivery, where limited leakage from liposomes while fusing to a target cell is desired. On the other hand, the insertion of a nucleobase at the C‐terminus didn't produce significant perturbing effects on the peptide‐membrane interaction, that is the main mechanism of trichogin antimicrobial activity.[31]

### 2.4 Dynamic Light Scattering (DLS)

It is known that positively charged peptides promote aggregation in negatively charged liposomes. DLS is a powerful tool to study aggregation/fusion of model vesicles.[32] We used it to study the phenomenon as a function of peptide concentration, presence/absence of nucleobases, and the number of basic residues in the sequence. Experiments have been carried out on SUVs of lipid composition POPE/POPG 7:3 (SUVs PEPG), in the presence of each peptide and at three P/L ratios. Results are summarized in Table 3.

We used the Z‐average size to compare our results since our samples were all measured in the same dispersant, by the same technique. The polydispersity (Polydispersity Index, PdI) was below 0.5 for all measurements, apart from those detecting very large particles (>10'000nm), where the results quality was, as expected, poor. The size distributions are reported in the Supporting Information.

We studied all our trichogin analogs, keeping the concentration of both peptide and lipids in the mother solutions constant. Starting from a pristine liposome with a hydrodynamic diameter of about 100 nm, we found a 100% increase in the presence of the reference peptide Oct‐K2569Tric‐Lol at P/L 1:15. At the same P/L ratio, the substitution of the naturally‐occurring C‐terminal amino alcohol with Thym resulted in a significant change in the dimension of the model vesicles, with a 300% increase. The peptide analogs containing two/three Lys residues in the sequence did not produce large vesicles at that P/L ratio. This observation most likely stems from their reduced possibility to neutralize the lipid negative charge, being less positive. By increasing the P/L ratio to 1:6, bigger aggregates of liposomes, with a diameter > 3000 nm, were detected for the analogs containing four Lys residues. A remarkable effect is observed when the high P/L ratio of 1:3 is reached. Peptide‐promoted aggregation is usually reversible [6], with disaggregation occurring on negatively charged liposomes at a high concentration of the cationic peptide, that makes the liposomes positively charged, thus inducing their separation for electrostatic repulsion [33]. This is indeed the case for the reference compound Oct‐K2569Tric‐Lol: at P/L 1:3, the disaggregation takes place, with a detected hydrodynamic diameter of about 100 nm, close to the value registered for the pristine liposomes and the disappearance of any visible turbidity. Conversely, the presence of a nucleobase seems to hamper the separation, resulting in even larger aggregates. Going to the highest P/L ratio tested, 1:2, the hydrodynamic diameter remains extremely high (Z‐averaged size about 10'000 nm, see Supporting Information) for the nucleobase‐containing peptides, while the value registered at P/L 1:2 for the reference peptide Oct‐K2569Tric‐Lol remains at about 100 nm, namely the average diameter of pristine PEPG SUVs. The peptide analogs containing two or three Lys residues in the sequence resulted in the formation of huge liposomes only at the highest P/L ratios tested (1:3 and 1:2). No reversibility was detected for those two analogs.

Peptide‐mediated aggregation of liposomes is a common observation with cationic peptides, arising from membrane surface neutralization upon peptide binding.[6, 34] The P/L ratio in which aggregation occurs should correspond approximately to electroneutrality. Considering a peptide charge of +4 (as in Oct‐K2569Tric‐Lol, Oct‐K2569Tric‐T, and T‐K2569Tric‐A) and 30% content of anionic POPG lipids in the membrane studied herein, the total charge of the membrane and the total charge of the peptides (at a 10 µM concentration) should be equal but opposite in the presence of 133 µM lipid, corresponding to a P/L ratio of 1/13. To follow the complete aggregation/disaggregation phenomenon, we thus performed the DLS analysis at three P/L ratios: 1/15 (below the theoretical eletroneutrality, thus before aggregation), 1/6, when electroneutrality is surely reached, thus aggregation should be visible, and 1/3, when the prevalence of positive charged peptides over negatively charged lipids should cause disaggregation, as reported in the literature [6, 34]—if electrostatic interactions are the main driving force for liposome aggregation. As shown in Table 3, our DLS results for Oct‐K2569Tric‐Lol seem to reflect the evolution described above, with peptide‐induced aggregation visible at P/L 1/6 and disaggregation occurring at higher P/L, highlighting the reversible nature of the process, and that therefore aggregation predominates on fusion for this peptide. Besides, the turbidity observed at lower P/L for Oct‐K2569Tric‐Lol disappeared at P/L 1/3. This is not the case for peptides Oct‐K2569Tric‐T and T‐K2569Tric‐A, also bearing four Lys residues. For those two peptides, not only the visible turbidity increase at high P/L ratios, but also higher hydrodynamic diameters than at 1/6 were detected by DLS. In summary, our DLS experiments suggest that in the case of the helical, cationic peptide Oct‐K2569Tric‐Lol (without Thymine), the aggregation process is reversible and strongly driven by electrostatic interactions, while in the presence of one or two nucleobases at the peptide termini, an irreversible, fusion process seems to occur. We conclude that the presence of a nucleobase is likely not enough to *induce* fusion in the absence of efficient electrostatic interaction, but rather it probably promotes irreversible membrane fusion over reversible liposome aggregation.

## 3 Transmission Electron Microscopy (TEM) Analysis

**FIGURE 3:**
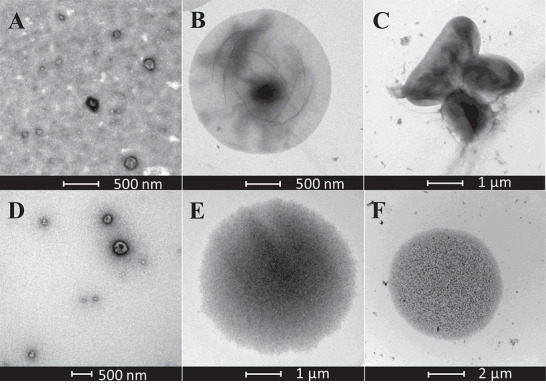
TEM images of: (**A**) Freshly made liposomes PE:PG 7/3. (**B**) The same liposomes after addition of **T‐K2569Tric‐A** P/L 1:2 (measured diameter: 1938 nm). (**C**) A particular of the liposome aggregation mediated by the peptide **T‐K2569Tric‐A** self‐assembled in molecular wires, highlighted by the Uranyl acetate staining. (**D**) The same liposomes after the addition of **Oct‐K2569Tric‐Lol** P/L 1:2. (**E**) The same liposomes after the addition of **Ac‐K2569Tric‐T**. (**F**) The same liposomes after addition of **Oct‐K2569Tric‐T** P/L 1:2, namely the maximum P/L tested. Final peptide concentration in all cases: 3×10^−4 ^M.

We performed TEM analysis on the same, freshly prepared samples analyzed by DLS. The samples were stained with Uranyl acetate to highlight the presence of the peptide. Figure 3 shows the comparison between pristine PEPG SUVs and the same liposomes treated with T‐K2569Tric‐A and the reference compound Oct‐K2569Tric‐Lol at the highest P/L ratio tested (1/2). More images are reported in Figures S43–S46, Supporting Information. Oct‐K56Tric‐T and Oct‐K259Tric‐T were not analyzed. TEM analysis confirmed that liposome aggregation promoted by Oct‐K2569Tric‐Lol is reversible, as expected (Figure 3D). Indeed, liposomes treated with Oct‐K2569Tric‐Lol at P/L 1:2 appear to be of the same diameter as the pristine SUVs (Figure 3A), while big vesicles are found in the sample containing T‐K2569Tric‐A (Figure 3B), Oct‐K2569Tric‐T (Figure 3D), and Ac‐K2569Tric‐T (Figure 3E) at the same P/L ratio.liposomi+ T‐pept‐Aliposomi+ T‐pept‐A Figure 3C reports the single example of liposomes aggregation we could detect for Thym‐bearing peptides: we consider this result as an indication that a balance between fusion and aggregation might be present, although largely shifted toward fusion: during our accurate TEM analysis in search of such examples, we could spot only this single one, over all the peptides analyzed, while all other observations included large vesicles like the one reported in Figure 3B. Overall, our TEM analysis confirms and adds experimental evidence to the DLS conclusions. Finally, T‐K2569Tric‐A was already reported as being able to form long, self‐assembled molecular wires, guided by nucleobase self‐recognition. Such wires are visible thanks to the Uranyl staining, for instance, in Figure 3C. Thymine‐Thymine H‐bond formation is well known and has been exploited several times, e.g., to create new materials [35]. The interaction between nucleobases probably plays an important role in promoting the observed liposome fusion.

**FIGURE 4:**
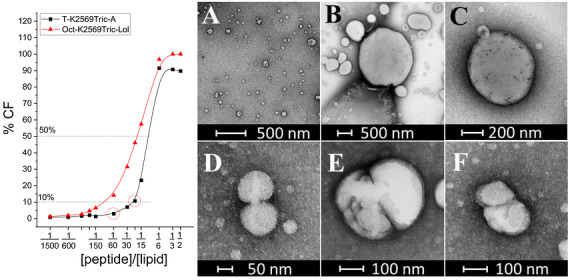
The circles highlight the percentages of CF leakage obtained at P/L 1:20 (10% leakage, corresponding to TEM panels B‐C) and 1:60 (leakage below 5%, corresponding to TEM panels D‐F) for **T‐K2569Tric‐A**. TEM images recorded for the pristine SUVs are reported in panel A.

We performed the TEM analysis also at lower P/L ratios, namely when limited percentages of leakage occur. Figure 4 shows the correlation between the level of leakage and the TEM images registered at the low P/L ratios of 1:60 and 1:20 for T‐K2569Tric‐A. Indeed, at those low P/L, we could detect peptide‐promoted membrane aggregation/fusion, resulting in large liposomes, while no such aggregates were found for the pristine PEPG SUVs.

### 3.1 Isothermal Titration Calorimetry (ITC)

**FIGURE 5:**
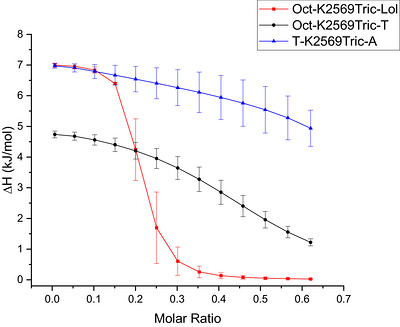
Integrated isotherm of the calorimetric titration of SUVs made of POPE/POPG 7:3 with the three peptides **Oct‐K2569Tric‐Lol** (red curve), **Oct‐K2569Tric‐T** (black curve), and **T‐K2569Tric‐A** (blue curve). The curves are averages (with error bars) of repeated independent measures.

The experimental data herein presented seem to indicate that derivatization of the cationic helical peptide scaffold K2569Tric with a single thymine nucleobase may be able to convert a reversible liposome aggregation process into an irreversible membrane fusion event. To investigate the thermodynamic origin of that switch in reversibility, we performed Isothermal titration calorimetry (ITC) analysis, aiming at measuring the enthalpy of the processes. To fully evaluate the effect of nucleobase derivatization, we analyzed the three peptides Oct‐K2569Tric‐Lol, Oct‐K2569Tric‐T, and T‐K2569Tric‐A, sharing the same net charge, in the P/L molar ratios range from 0 to 0.6 (namely from 1/18 to 1/1.6). The experimental procedure consisted in the injection of 3 µL aliquots of each peptide at a concentration of 3.33×10^−4^ M into a solution of POPE/POPG 7:3 liposomes (SUVs) at an initial concentration of 1.0×10^−4^ M. We repeated the measurement four times for each peptide, at two reference power settings (5 and 10 μcal/sec, respectively), to ensure reproducibility (see Figures S38–S40, Supporting Information). The mean normalized injection enthalpy curve of the process for each peptide is reported in Figure 5 as a function of P/L ratio, along with the error bars. Raw traces are reported in Figures S38–S40, Supporting Information. In all cases, the titrations resulted in positive peaks, confirming the endothermic character of liposome aggregation/fusion phenomena already described in the literature.[28, 36]. The endothermic nature of liposome‐liposome aggregation/fusion is explained in the literature by the presence of energetic barriers to be overcome, due to the need of bringing the membranes in close contact and, for fusion, modifying the local curvatures of the lipid layers [37, 38]. The results for Oct‐K2569Tric‐Lol highlight a clear change in enthalpy as a function of P/L ratio, starting approximately at P/L 0.15 (1/7), corresponding to the P/L at which disaggregation should begin, with the endothermic heat decreasing to zero (at P/L 0.3, 1/3.3) as the P/L ratio increases. Considering the results in the framework of those obtained by DLS and TEM analysis, we tend to attribute this decrease in the endothermic heat to the onset and progression of liposomes disaggregation, a process that does not require significant heat input to occur [39]. In contrast, no such effect is observed for Oct‐K2569Tric‐T, and T‐K2569Tric‐A. For those two peptides, such a sharp decrease in the enthalpy heat was not observed, pointing to an endothermic fusion process that continues to occur also at high P/L ratios, in agreement with the parallel increase in the hydrodynamic diameter observed with DLS. We hypothesize that T–T self‐association (weaker than Watson–Crick) may be able to offer multivalent contact zones across the membrane. This would explain also the ITC results for T‐K2569Tric‐A, where Watson‐Crick A‐T seems thermodynamically performing better than Oct‐K2569Tric‐T. Hydrophobic stacking at the bilayer interface may also contribute by stabilizing close membrane apposition and displacing structured interbilayer water, thus reducing hydration repulsion. The endothermal enthalpies of the processes of aggregation and fusion, obtained by fitting the isothermal curves, were all in the range 5–7 kJ/mol: 7.1±1.1, 5.2±1.7, and 7.2±1.3 kJ/mol, respectively, compatible with the endothermic energy values reported in the literature.[28, 36] Taken together, the ITC results seem to confirm that the presence of a nucleobase functionalization modifies the peptide‐membrane interaction, supporting our view of a switch from peptide‐induced reversible aggregation of liposomes to an irreversible fusion process.

## 4 Conclusions

Peptides that trigger membrane aggregation can be used to create new peptide/lipid nanostructures or co‐aggregates for various applications. These novel assemblies could have roles in diagnostics, drug delivery, or as components in bio‐inspired materials. In this work, we synthesized several analogs bearing a nucleobase at their N‐ and/or C‐terminus and proved them against negatively charged small unilamellar vesicles (SUVs). By combining leakage experiments, dynamic light scattering, transmission electron microscopy, isothermal titration calorimetry, and circular dichroism studies, we demonstrated that the presence of the nucleobase can promote peptide‐mediated liposome irreversible aggregation/fusion. Oct‐K2569Tric‐Lol, containing four Lys residues, was found to induce reversible liposome aggregation, while the derivatization of the sequence to include one or two nucleobases at the peptide termini resulted in the irreversible formation of large vesicles. The presence of complementary nucleobases at both peptide termini also promotes membrane fusion, even at peptide‐to‐lipid ratios devoid of leakage activity. Peptides that interact with lipid bilayers are crucial for many biological processes. The possibility to enhance the ability of cationic peptides to promote membrane aggregation/fusion herein described, might be useful to create new tools to trigger targeted drug release from liposomes, control membrane fusion, enhance antimicrobial activity, and develop novel diagnostic or therapeutic agents for various diseases by manipulating cellular processes involving lipid membranes.

## 5 Experimental

### 5.1 Synthesis and Characterizations

The newly synthesized trichogin analogs were synthesized by manual solid‐phase peptide synthesis in good yield (from 45% to 81%) and purity (≥94%) after purification. Double couplings were performed for all steps involving Aib residues. Coupling reagents were the green Oxyma pure and diisopropylcarbodiimide (DIC) in the solvent mixture Ethyl Acetate (EtOAc)/Dimethylsulfoxide (DMSO) 4:1. Fluorenylmethyloxycarbonyl (Fmoc) protecting group was used as backbone protection, removed by two treatments with Piperidine 20% in *N,N*‐dimethylformamide (DMF) (5 and 10 min, respectively). Washings were performed after deprotection, with EtOAc/DMSO 5:1. The sequences were grown over a 2‐chlorotrytil resin preloaded with 1,2‐aminoalcohol Leucinol and cleaved from the resin under mild conditions (HFIP) 30% in CH_2_Cl_2_). Thym or Ade were added in solution with EDC*/*HOBt, to avoid the presence of Oxyma Pure and DIC, which complicate the purification step. Work‐up comprised liquid‐liquid separation with acidic (KHSO_4_ 5% in H_2_O) and distilled water (3 washings each), anhydrification over Na_2_SO_4_, to result in the crude fully protected peptides. Purification of each peptide at that stage, to ≥ 94% purity, was performed by medium‐pressure reverse‐phase chromatography (Biotage Isolera Prime instrument), gradient 0%–100%B (A: H_2_O, N: Acetonitrile (ACN)/H_2_O 9:1 + HCl 0.01 M) followed by 100% methanol. The peptides were obtained in this final solvent, avoiding the need of freeze‐drying them.

Lys protecting group *tert*Butyloxycarbonyl (Boc) was removed after peptide release from the solid support and purification, by treating the purified, fully protected peptide with HCl 3 M in methanol for 1 h. Pure peptides were obtained by precipitation from ethyl ether. Peptide characterizations of each peptide by high‐resolution mass spectrometry and 1D NMR are reported in Supporting Information. The NMR experiments were performed at 298 K with a Bruker *AVANCE DRX‐*400 instrument operating at the frequency of 400 MHz for protons.

### 5.2 Circular Dichroism

The circular dichroism (CD) measurements were carried out in distilled water or in the presence of PEPG liposomes (obtained as described below for the leakage experiments) on a Jasco (Tokyo, Japan) model J‐715 spectropolarimeter at room temperature, using Hellma (Müllheim, Germany) quartz cells with Suprasil windows and optical path length of 1, 0.1, or 0.02 cm. The signal‐to‐noise ratios were improved by accumulating 16/32 scans. Data were processed using the J‐700 program for Windows and expressed in terms of [Θ]_T_, the total molar ellipticity (deg × cm^2^ × dmol^−1^).

### 5.3 Dynamic Light Scattering (DLS)

The Light Scattering spectroscopy was performed on a Malvern ZS nano DLS and Zeta potential equipment, which allows to determine the dimensions of dissolved and suspended particles, under controlled temperature of the sample compartment. Zeta Potential was measured by electrophoretic light scattering from particles.

### 5.4 Leakage Experiments

1‐palmitoyl‐2‐oleoyl‐sn‐glycero‐3‐phosphoethanolamine (POPE) and 1‐Palmitoyl‐2‐oleoyl‐sn‐glycero‐3‐phosphoglycerol (POPG) were purchased from Avanti Polar Lipids, Inc. (Alabaster, AL). The lipid mixture PE/PG (7 : 3) was dissolved in CHCl_3_ in a test tube, dried under N_2_, and lyophilized overnight. The lipid film was reconstituted with a solution of carboxyfluorescein (CF) in 30 mM Hepes buffer (pH 7.4) at room temperature for 1 h. To make SUVs, the resulting multilamellar vesicle suspension was sonicated (GEX 400 Ultrasonic Processor, Sigma) on ice until the initially cloudy lipid dispersion became translucent. The excess of fluorescent dye was eliminated by gel filtration on Sephadex G‐75 (Sigma). SUVs were diluted to a concentration of 0.06 mM with Hepes buffer (5 mm Hepes, 100 mM NaCl, pH 7.4). The SUVs were stored at 4°C and used within 24 h. The peptide‐induced leakage from SUVs was measured at 293 K on a Perkin Elmer model MPF‐66 spectrofluorimeter. The phospholipid concentration was kept constant (0.06 mm), and increasing P/L molar ratios were obtained by adding aliquots of water solutions of peptides, except for the native nonhydrosoluble trichogin GA IV, used as a reference compound, which was added as a methanol solution, keeping the final methanol concentration below 5% by volume. After rapid and vigorous stirring, the time course of fluorescence change corresponding to CF escape was recorded at 520 nm (6‐nm band pass) with *λ*_exc_ 488 nm (3‐nm band pass). The percentage of released CF at time *t* was determined as (*F*_t_−*F*_0_)/(*F*_T_−*F*_0_) x 100, with *F*_0_ = fluorescence intensity of vesicles in the absence of peptide, *F*_t_ = fluorescence intensity of vesicles at time *t* in the presence of peptide, and *F*_T_ = total fluorescence intensity determined by disrupting the vesicles by addition of 50 µL of a Triton X‐100 solution. The kinetics experiments were stopped at 20 min.

### 5.5 Transmission Electron Microscopy (TEM)

Samples were fixed with 2.5% glutaraldehyde in 0.1 M sodium cacodylate buffer, pH 7.4, overnight at 4°C, and postfixed with 1% osmium tetroxide plus potassium ferrocyanide 1% in 0.1 M sodium cacodylate buffer for 1 h at 4°C. After three water washes, samples were dehydrated in a graded ethanol series and embedded in an epoxy resin (Sigma‐Aldrich). Ultrathin sections (60‐70 nm) were obtained with an Ultratome Leica Ultracut EM UC7 ultramicrotome, counterstained with uranyl acetate and lead citrate, and viewed with a Tecnai G^2^ (FEI) transmission electron microscope operating at 100 kV. Images were captured with a Veleta (Olympus Soft Imaging System) digital camera.

### 5.6 Isothermal Titration Calorimetry (ITC)

ITC experiments were performed on a Microcal PEAQ‐ITC instrument (Malvern Panalytical, Malvern, UK). Both POPE/POPG SUVs (100 µM) and peptides (Oct‐K2569Tric‐Lol, Oct‐K2569Tric‐T, and T‐K2569Tric‐A, 333 µM each) were dissolved in Hepes buffer (5 mm Hepes, 100 mM NaCl, pH 7.4). Liposomes were contained in the measuring cell, and the peptide was injected via a Hamilton syringe. Titrations of the liposomes with each peptide were carried out at 20 °C. In each experiment, an initial 0.4 µL injection (excluded from subsequent data analysis) was followed by 12 independent injections of 3.0 µL with a stirring rate of 750 rpm to ensure rapid mixing. A 120 s interval between injections was applied to guarantee the equilibrium at each titration point. Blank experiments (liposomes against buffer) were carried out and subtracted to corresponding titrations in order to screen dilution heat contributions. Data were analyzed using the MicroCal PEAQ‐ITC Evaluation software (Malvern Panalytical, Malvern, UK). Integrated heat signals were fitted to “one set of sites” binding models. Values for the enthalpy change (ΔH) were obtained from curve fitting. All experiments were repeated twice per each reference power setting applied (5 and 10 μcal/sec, respectively, Figures S38–S40, Supporting information).

## Conflicts of Interest

All authors declare no conflicts of interest.

## Supporting information




**Supporting File**: chem71026‐sup‐0001‐SuppMat.pdf.
