# Supplementary material for: C‐Terminal Nucleobase Modification Amplifies Peptide‐Mediated Liposome Fusion
Source: Chemistry. 2026 Apr 22;32(26):e03665. doi: 10.1002/chem.202503665 (PMC13356288; doi:10.1002/chem.202503665)
Supplement: Supplementary file 1 — Supporting File: chem71026‐sup‐0001‐SuppMat.pdf. [file CHEM-32-e03665-s001.pdf]

Supporting Information

Title: **C-Terminal Nucleobase Modification Amplifies Peptide-Mediated Liposome Fusion**

Authors: **Laura Morbiato, Giacomo Bettin, Chiara Dalla Torre, Lorenzo Stella, Marta De Zotti***

INDEX

- Synthesis and Characterizations. p. S3

- DLS p. S13
- ITC p. S27
- CD p. S30
- TEM p. S33

Synthesis and Characterizations. In this work, we synthesized three novel trichogin GA IV analogs (Table S1). Characterizations were performed by High Resolution Electron Spray Ionization Mass Spectrometry (ESI-MS) analysis, and both ^{1}H and ^{13}C 1D NMR (Figures S1-S16).

Table S1. New peptide sequences synthesized in this work. The sequence of the naturally-occurring peptide trichogin GA IV is reported for comparison.

Acronym	Sequence[a]		
Trichogin GA IV[b]	*Oct-*	*Aib- Gly-Leu-Aib-Gly-Gly-Leu-Aib-Gly-Ile-Lol*	
Oct-TricK56-T	Oct-	Aib-Gly-Leu-Aib-Lys-Lys-Leu-Aib-Gly-Ile-Leu-	NH-$(CH_2)_2$-NH-Thym
Oct-TricK259-T	Oct-	Aib-Lys-Leu-Aib-Lys-Gly-Leu-Aib-Lys-Ile-Leu-	NH-$(CH_2)_2$-NH-Thym
Oct-TricK2569-T	Oct-	Aib-Lys-Leu-Aib-Lys-Lys-Leu-Aib-Lys-Ile-Leu-	NH-$(CH_2)_2$-NH-Thym
Ac-TricK2569-T	Ac-	Aib-Lys-Leu-Aib-Lys-Lys-Leu-Aib-Lys-Ile-Leu-	NH-$(CH_2)_2$-NH-Thym

[a]Thym, thymine-1-acetyl; Aib, α-aminoisobutyric acid; Ac, acetyl; Oct, *n*-Octanoyl; Lol, 1,2-aminoalcohol Leucinol. All amino acid residues are L. [b]The sequence of the naturally-occurring peptaibol trichogin GA IV is reported for comparison.

i. **Oct-TricK56-T**. Yield: 45%. Purity: 95%.

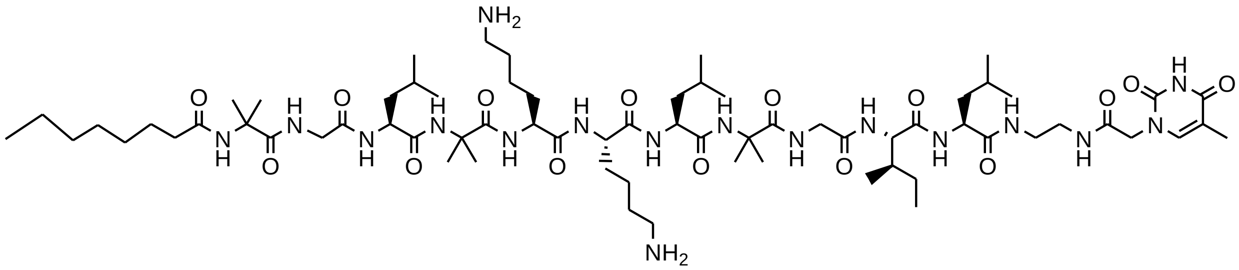

$C_{69}H_{123}N_{17}O_{15}$
Exact Mass: 1429.9385

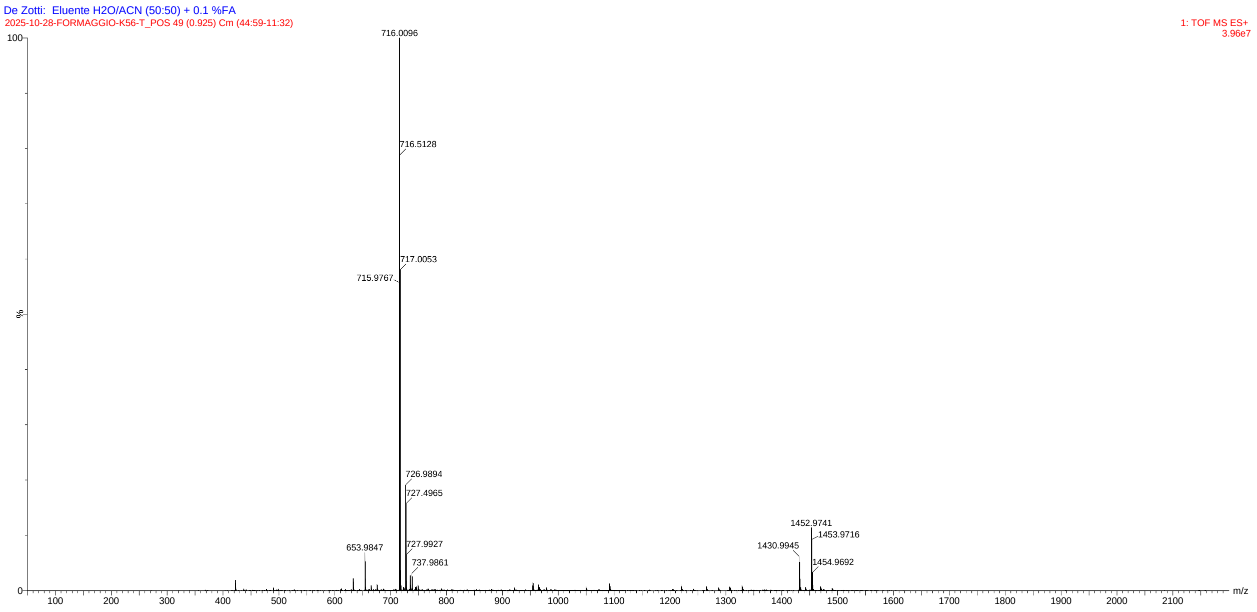

Figure S1. ESI-MS spectrum of Oct-TricK56-T. $[M+H]^{+}_{calcd.}$: 1430.9385 $[M+H]^{+}_{found}$: 1430.9945; $[M+Na]^{+}_{found}$: 1452.9741.

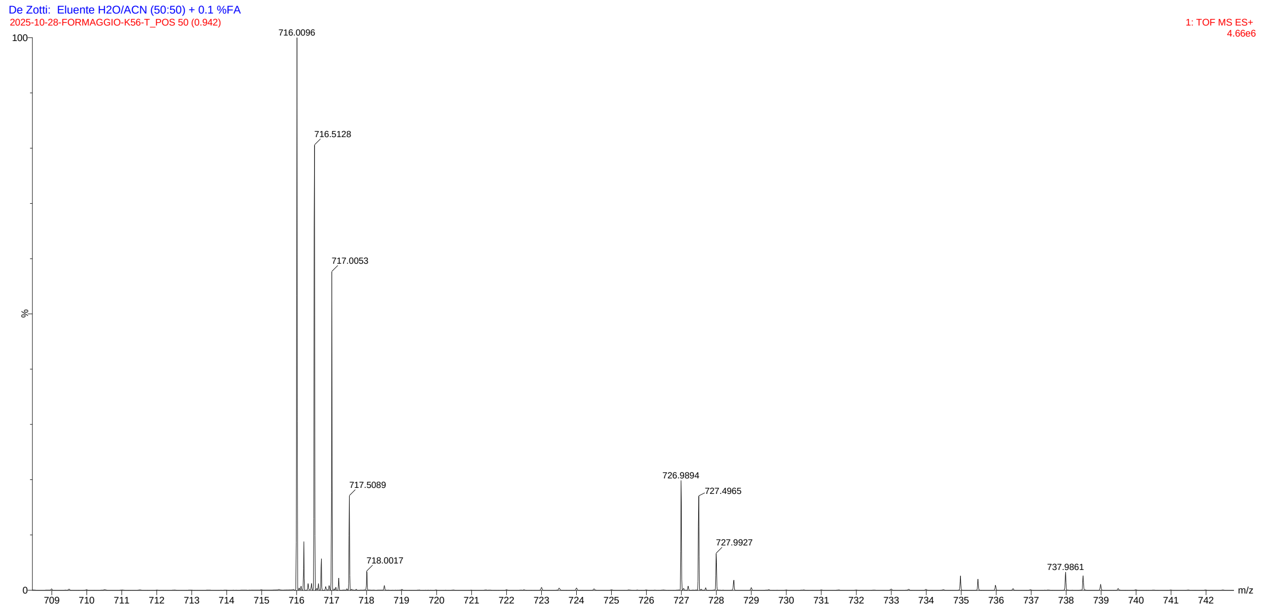

Figure S2. Detail of the ESI-MS spectrum of Oct-TricK56-T. $[M+H]^{+}_{calcd.}$: 1430.9385 . $[M+2H]^{2+}_{calcd.}$: 715.9693; $[M+2H]^{2+}_{found}$: 716.0096. $[M+H+Na]^{2+}_{calcd.}$: 726.9693; $[M+H+Na]^{2+}_{found}$: 726.9894; $[M+2Na]^{2+}_{found}$: 737.9661.

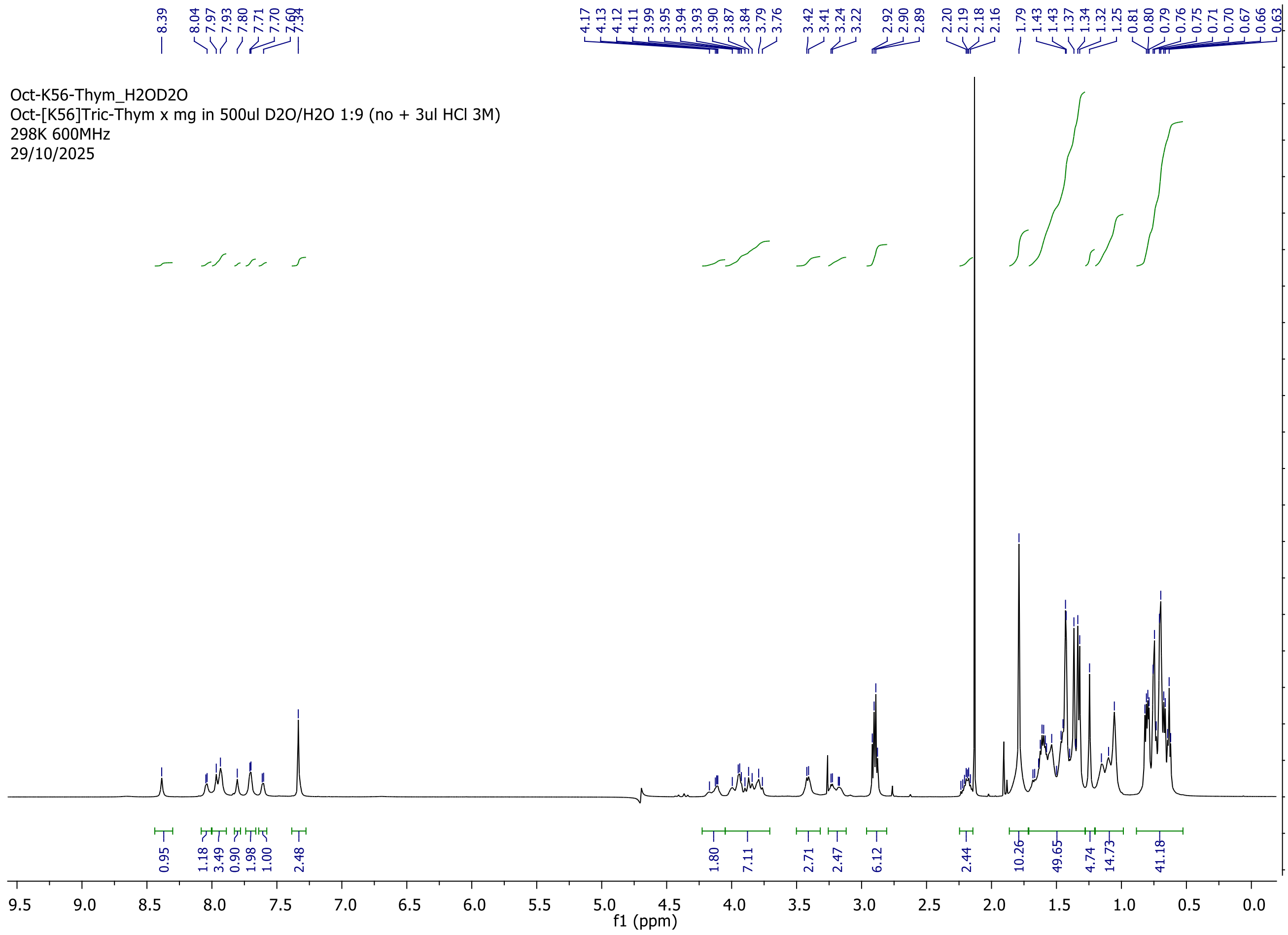

Figure S3. 1D ^{1}H NMR spectrum of Oct-TricK56-T in H_2O/D_2O 9:1 (600 MHz, 298K). Peptide concentration: 2mM.

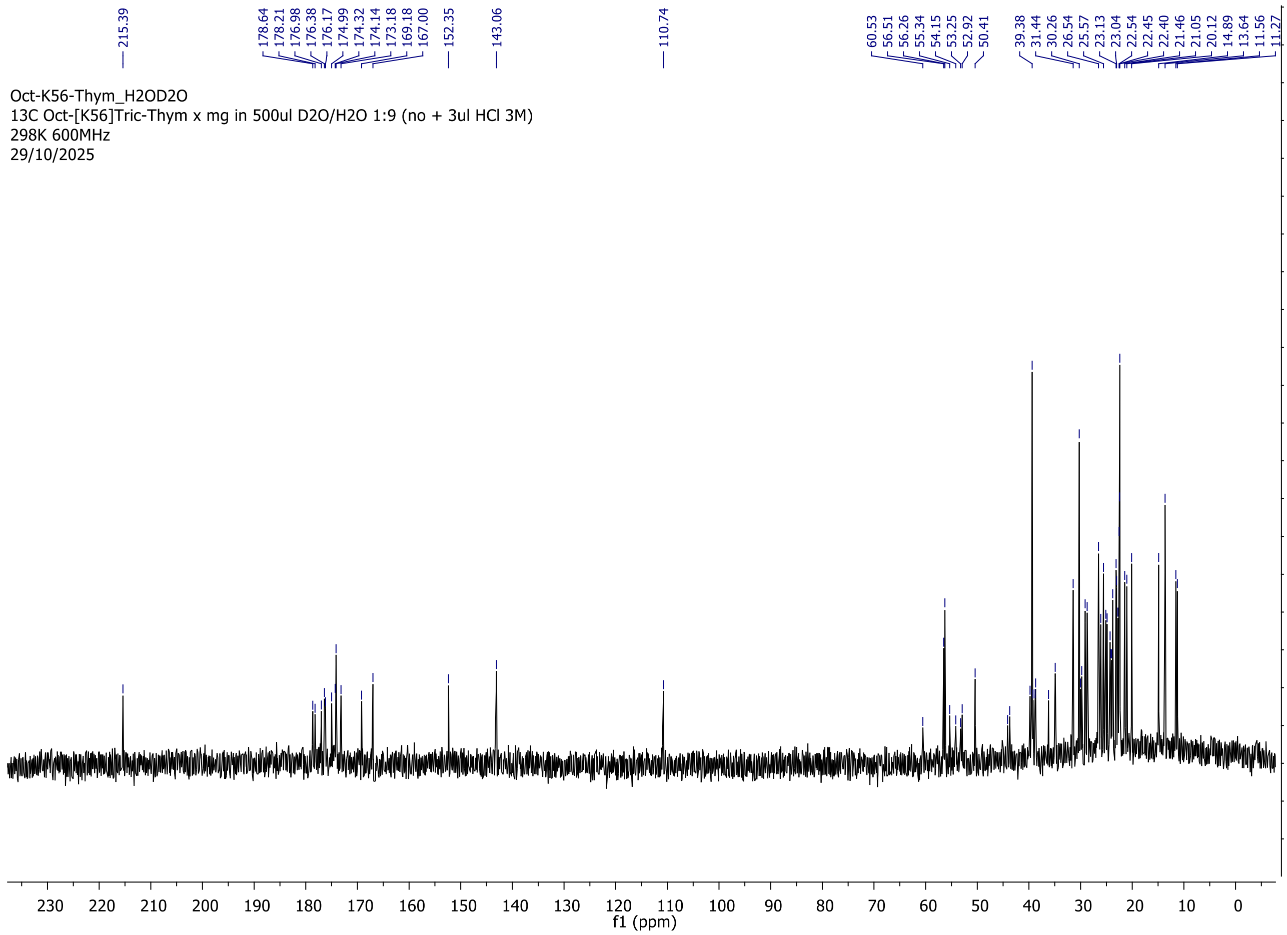

Figure S4. 1D ^{13}C NMR spectrum of Oct-TricK56-T in H_2O/D_2O 9:1 (150 MHz, 298K). Peptide concentration: 2mM.

ii. **Oct-TricK259-T**. Yield: 81%. Purity grade (after purification): 97%.

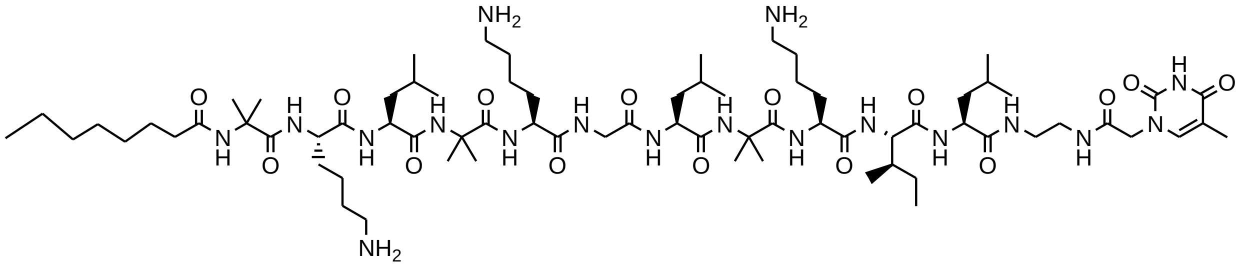

$C_{73}H_{132}N_{18}O_{15}$
Exact Mass: 1501.012

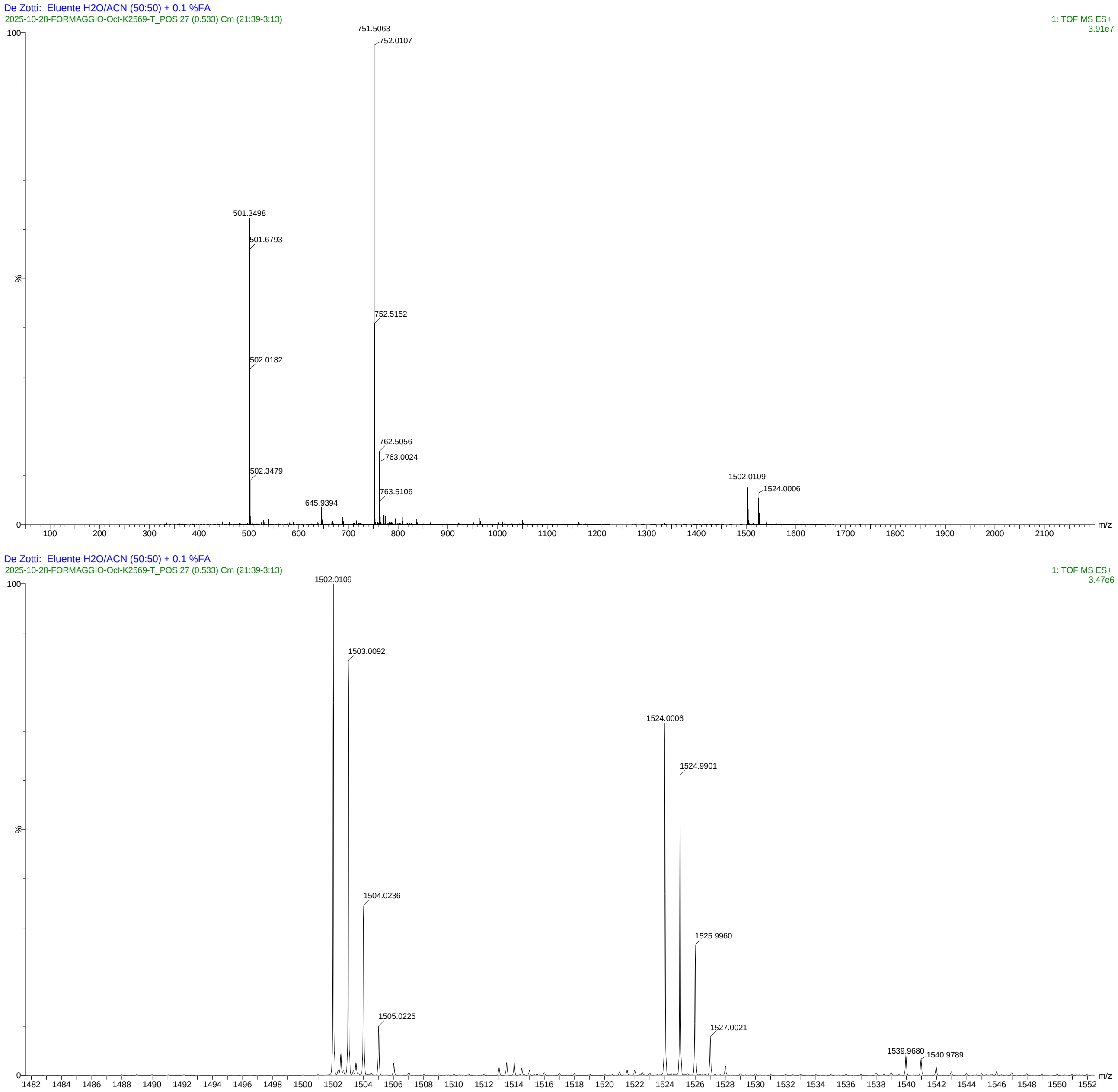

Figure S5. ESI-MS spectrum of Oct-TricK259-T. $[M+H]^+_{calcd.}$: 1502.012 $[M+H]^+_{found}$: 1502.0109; $[M+Na]^+_{found}$: 1524.0006; $[M+K]^+_{found}$: 1539.9680.

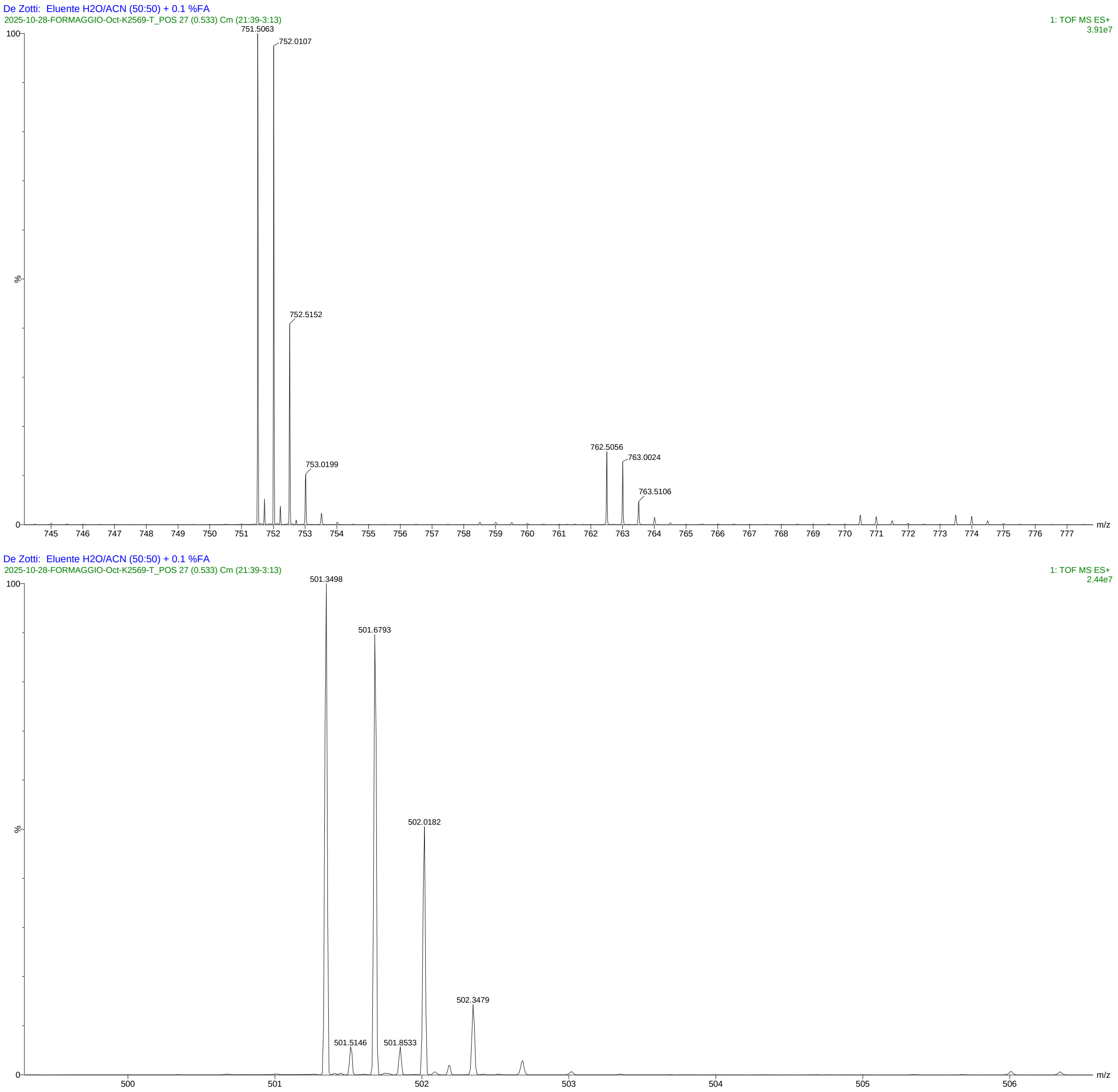

Figure S6. Details of the ESI-MS spectrum of Oct-TricK259-T. $[M+H]^{+}_{calcd.}$: 1502.0120. $[M+2H]^{2+}_{calcd.}$: 751.5060; $[M+2H]^{2+}_{found}$: 751.5063. $[M+H+Na]^{2+}_{calcd.}$: 762.5060; $[M+4H]^{4+}_{found}$: 762.5056.$[M+3H]^{3+}_{calcd.}$: 501.3373; $[M+3H]^{3+}_{found}$: 501.3498.

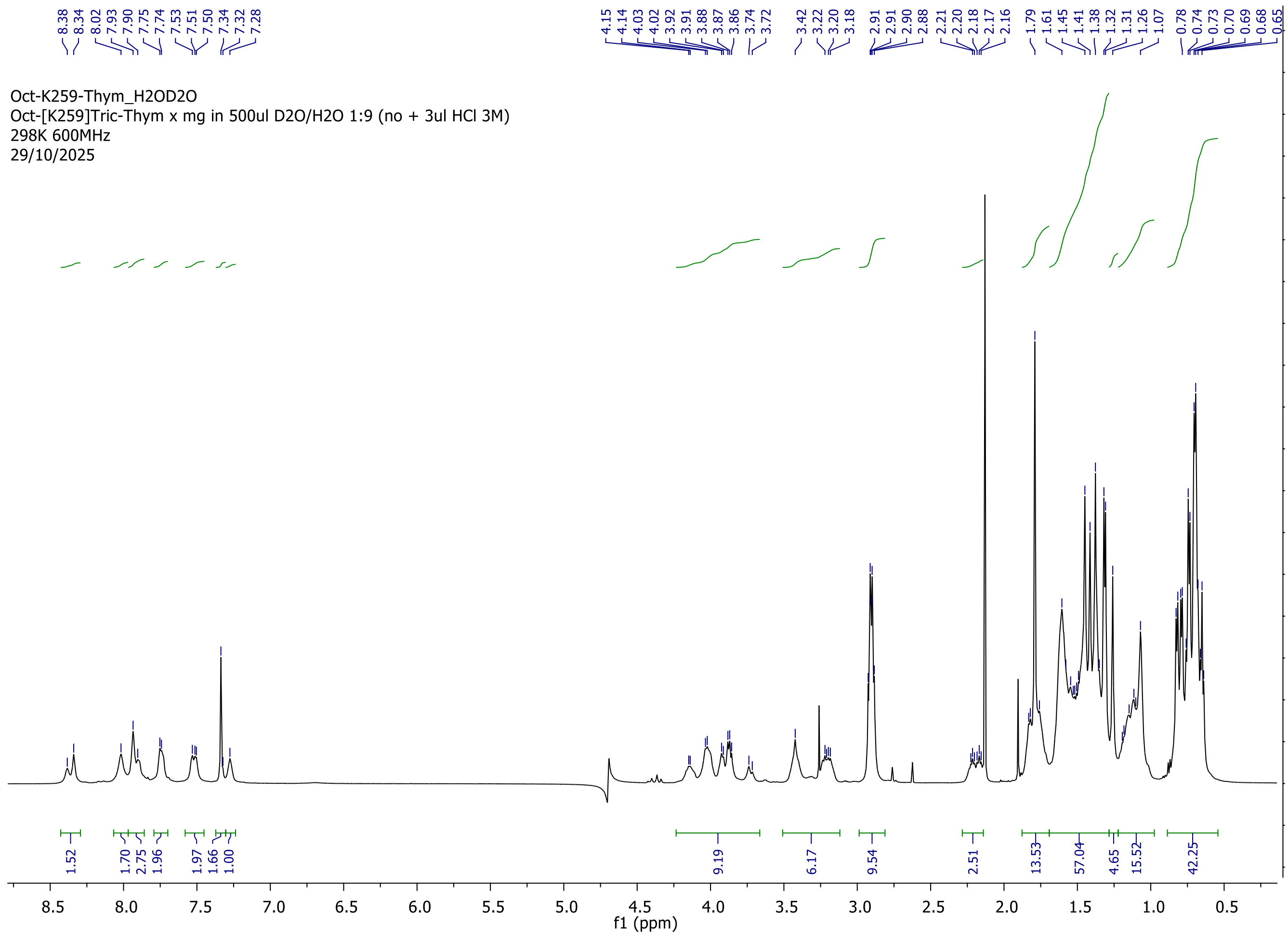

Figure S7. 1D ^{1}H NMR spectrum of Oct-TricK259-T in H_2O/D_2O 9:1 (600 MHz, 298K). Peptide concentration: 2mM.

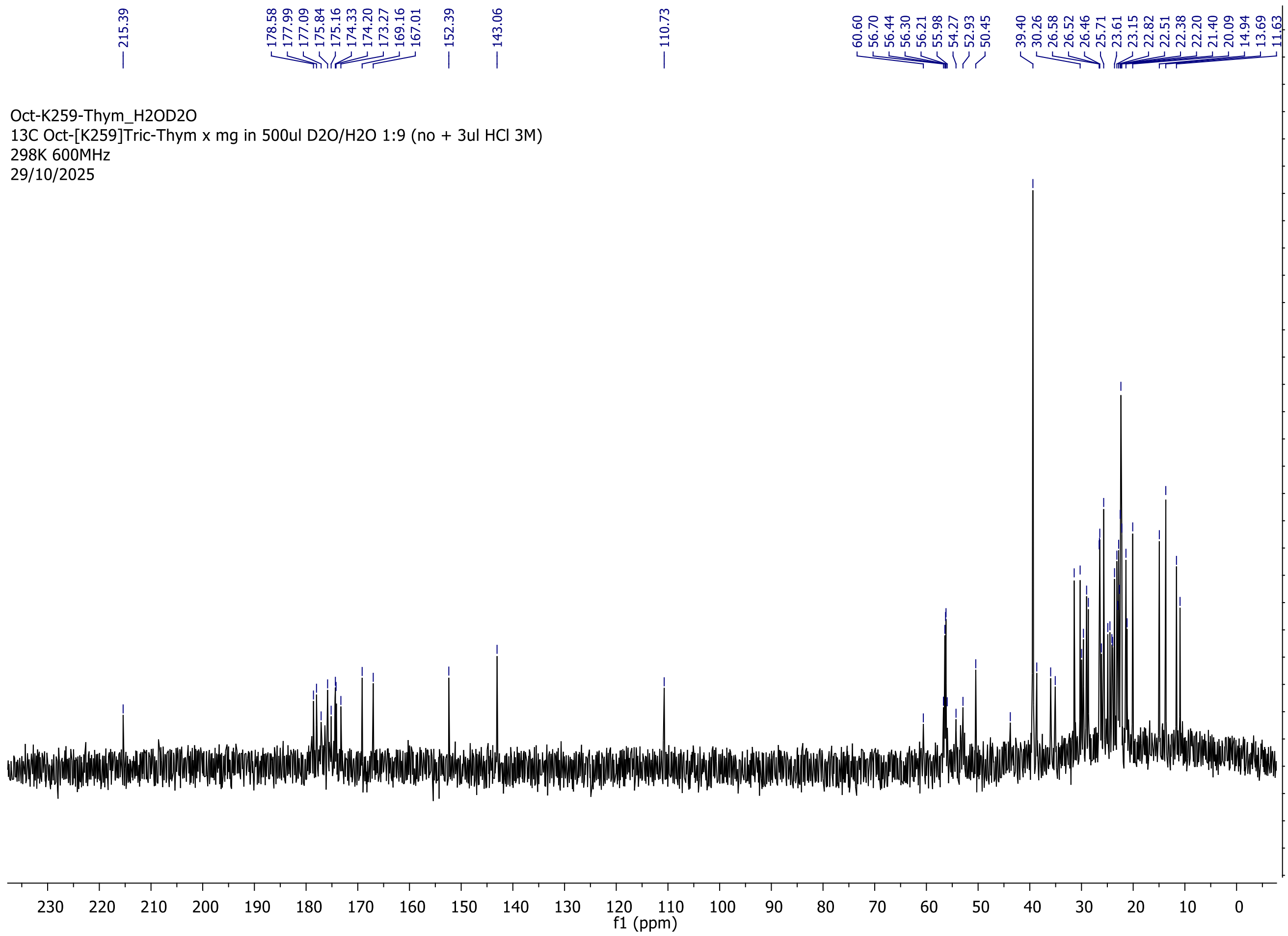

Figure S8. 1D ^{13}C NMR spectrum of Oct-TricK259-T in H_2O/D_2O 9:1 (150 MHz, 298K). Peptide concentration: 2mM.

iii. **Oct-TricK2569-T**. Yield: 75%. Purity grade (after purification): 95%.

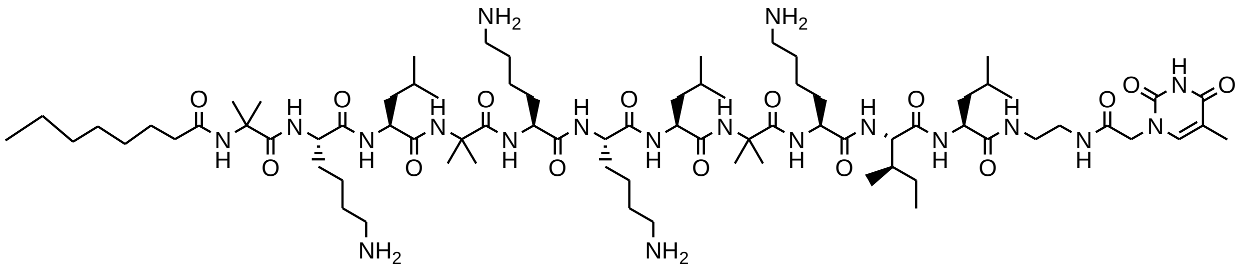

$C_{77}H_{141}N_{19}O_{15}$
Exact Mass: 1572.0855

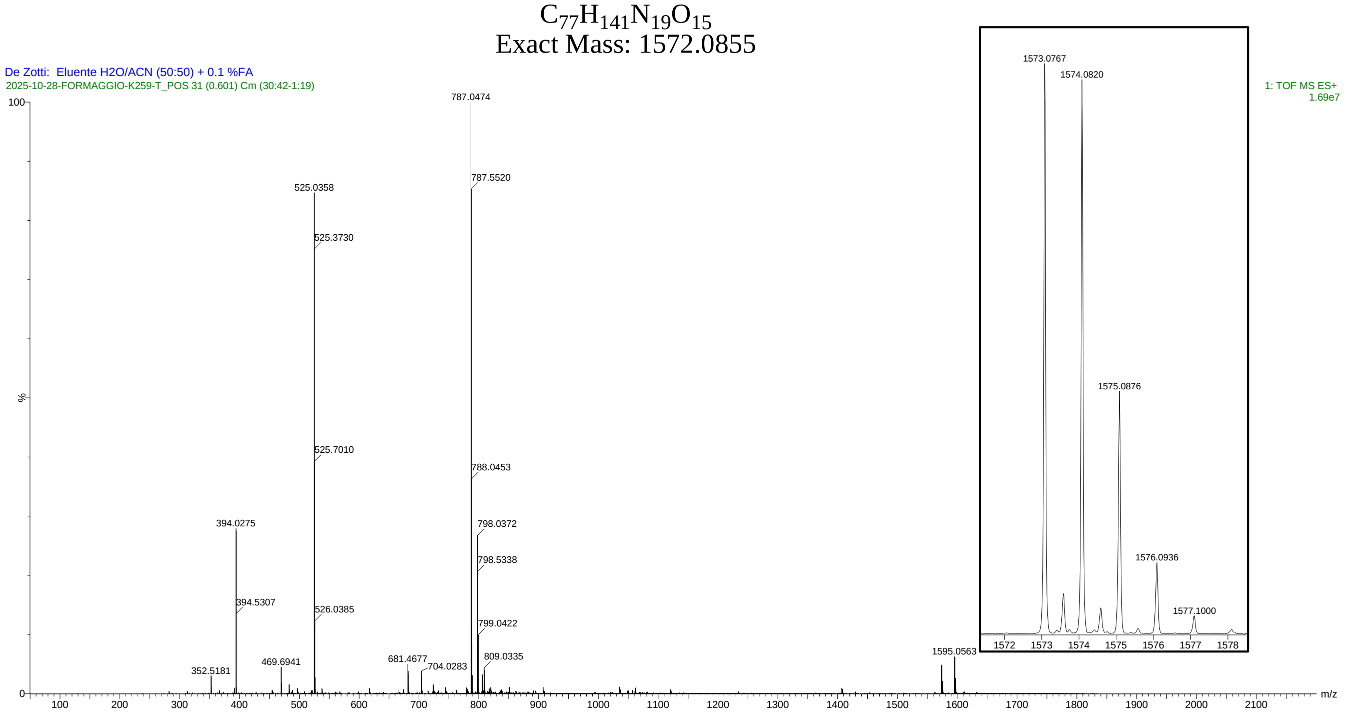

Figure S9. ESI-MS spectrum of **Oct-TricK2569-T**. $[M+H]^{+}_{calcd.}$: 1573.0855 $[M+H]^{+}_{found}$: 1573.0767; $[M+Na]^{+}_{found}$: 1595.0563. $[M+2H]^{2+}_{calcd.}$: 787.0450; $[M+2H]^{2+}_{found}$: 787.0474. $[M+H+Na]^{2+}_{calcd.}$: 798.0427; $[M+H+Na]^{2+}_{found}$: 798.0372. $[M+2Na]^{2+}_{calcd.}$: 809.0427; $[M+2Na]^{2+}_{found}$: 809.0335. $[M+3H]^{3+}_{calcd.}$: 525.03; $[M+3H]^{3+}_{found}$: 525.0358. $[M+4H]^{4+}_{calcd.}$: 394.02; $[M+4H]^{4+}_{found}$: 394.0275.

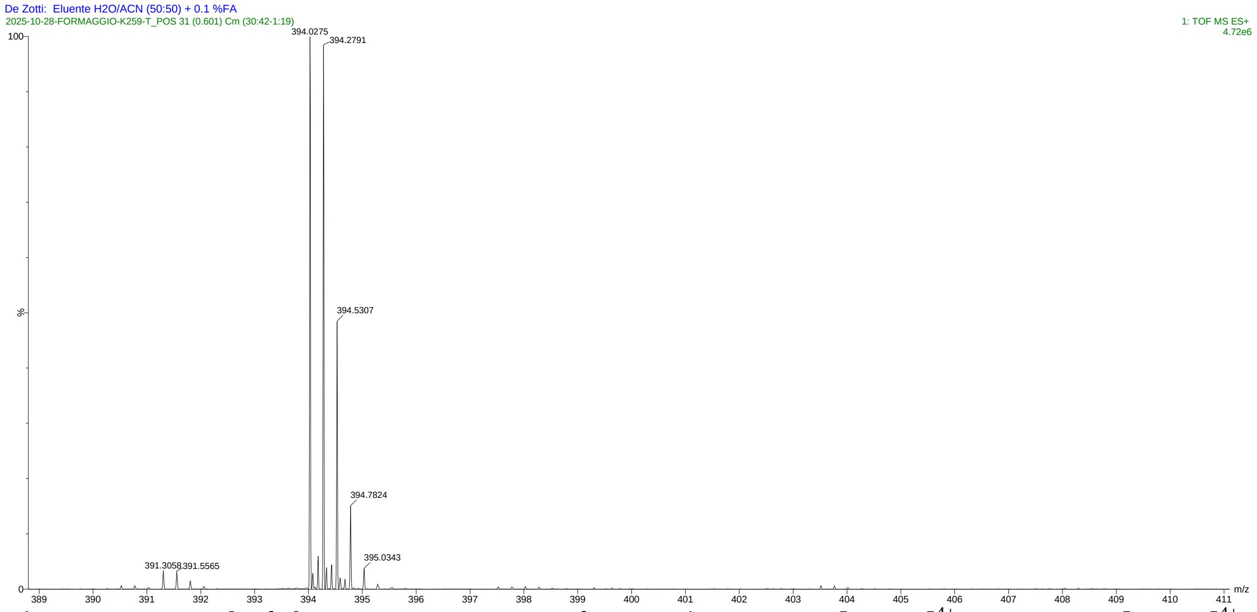

Figure S10. Detail of the ESI-MS spectrum of **Oct-TricK2569-T**. $[M+4H]^{4+}_{calcd.}$: 394.02; $[M+4H]^{4+}_{found}$: 394.0275.

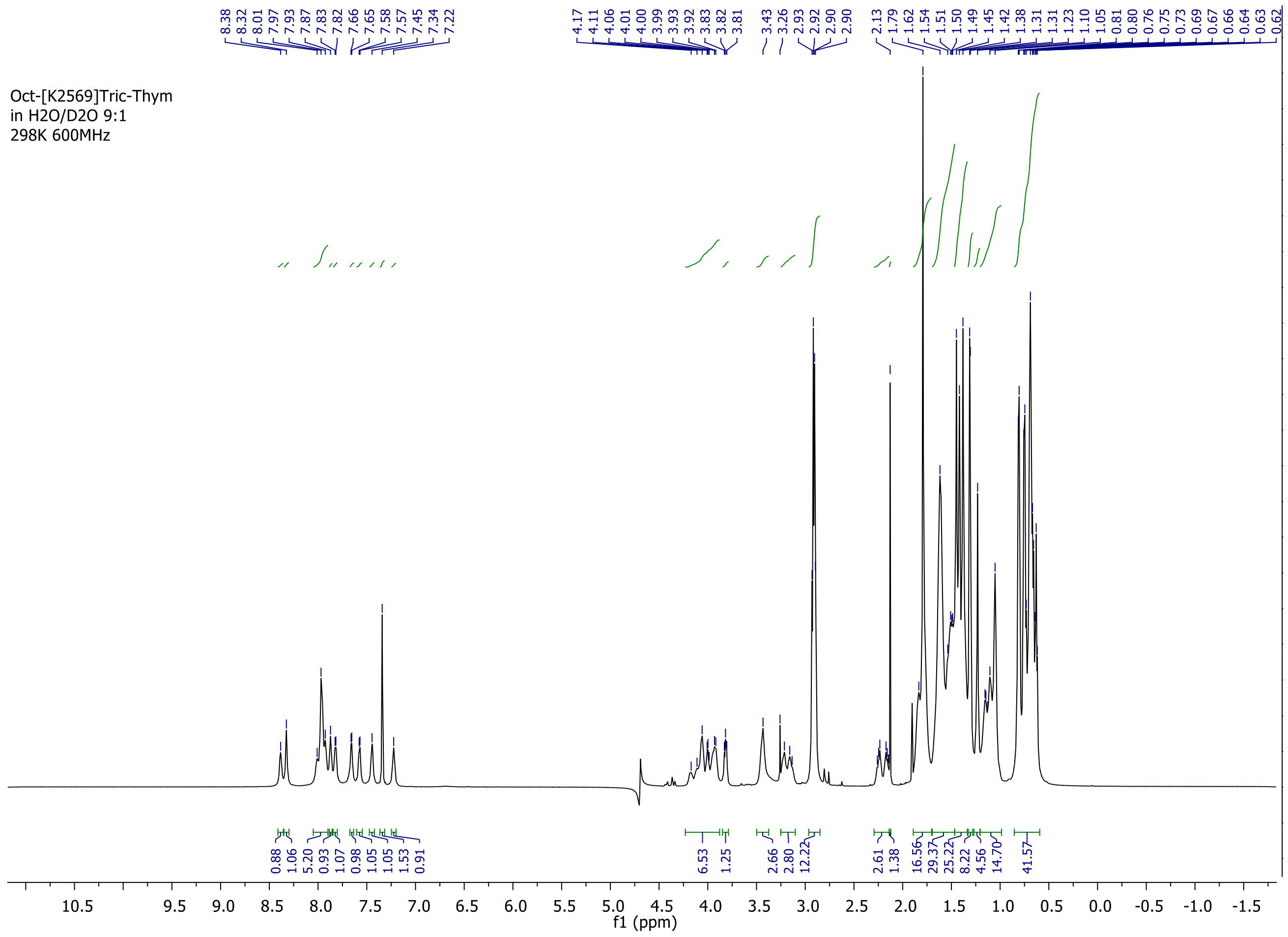

Figure S11. 1D ^{1}H NMR spectrum of Oct-TricK2569-T in H_2O/D_2O 9:1 (600 MHz, 298K). Peptide concentration: 2mM.

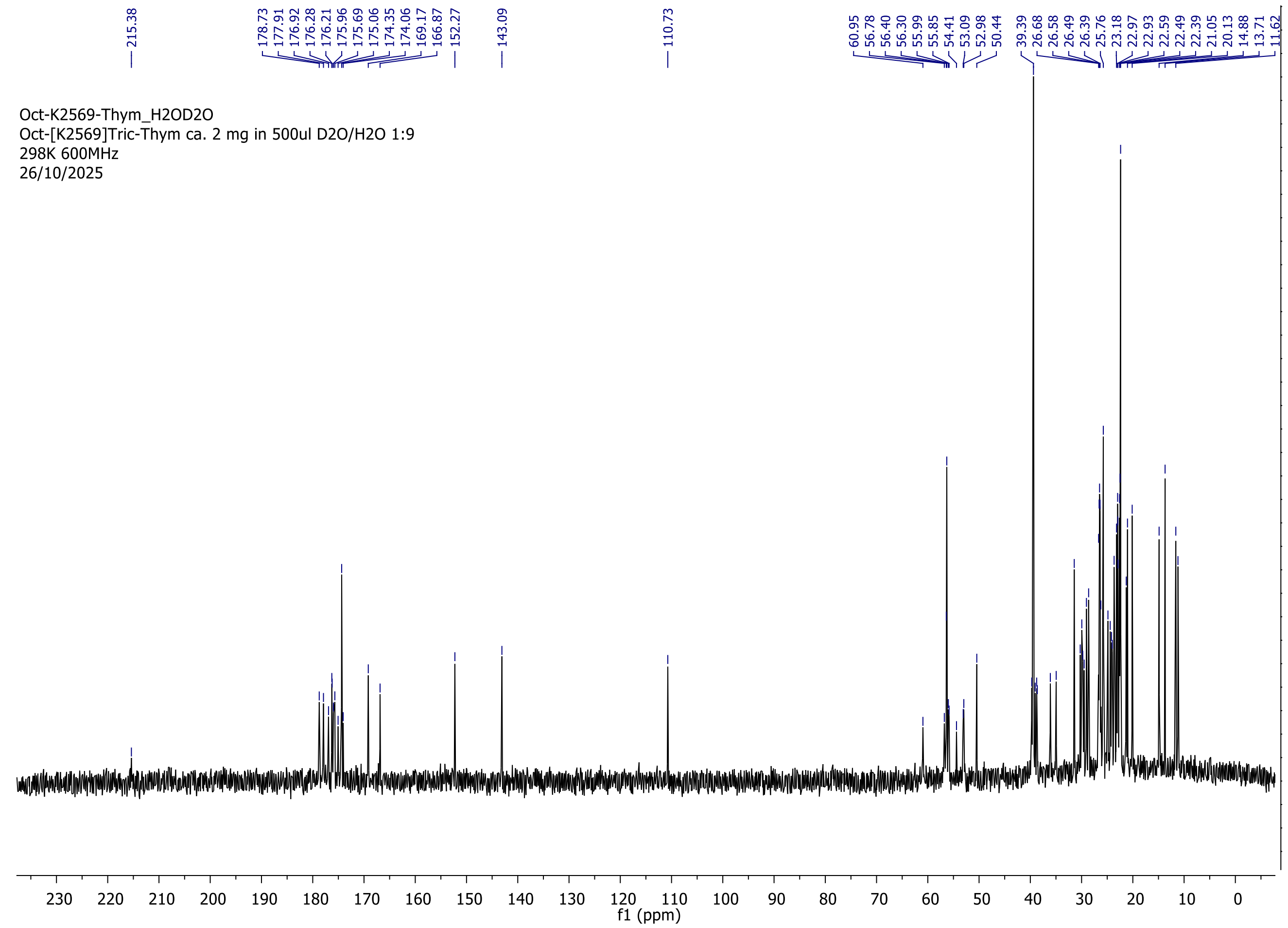

Figure S12. 1D ^{13}C NMR spectrum of Oct-TricK2569-T in H_2O/D_2O 9:1 (150 MHz, 298K). Peptide concentration: 2mM.

iv. **Ac-TricK2569-T**. Yield: 78%. Purity grade (after purification): 94%.

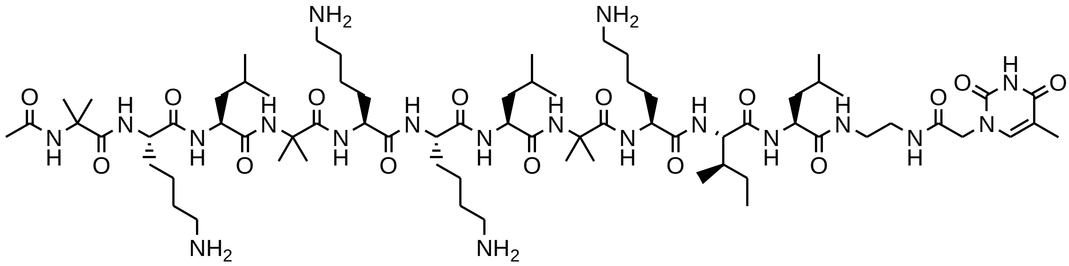

$C_{71}H_{129}N_{19}O_{15}$
Exact Mass: 1487.9916

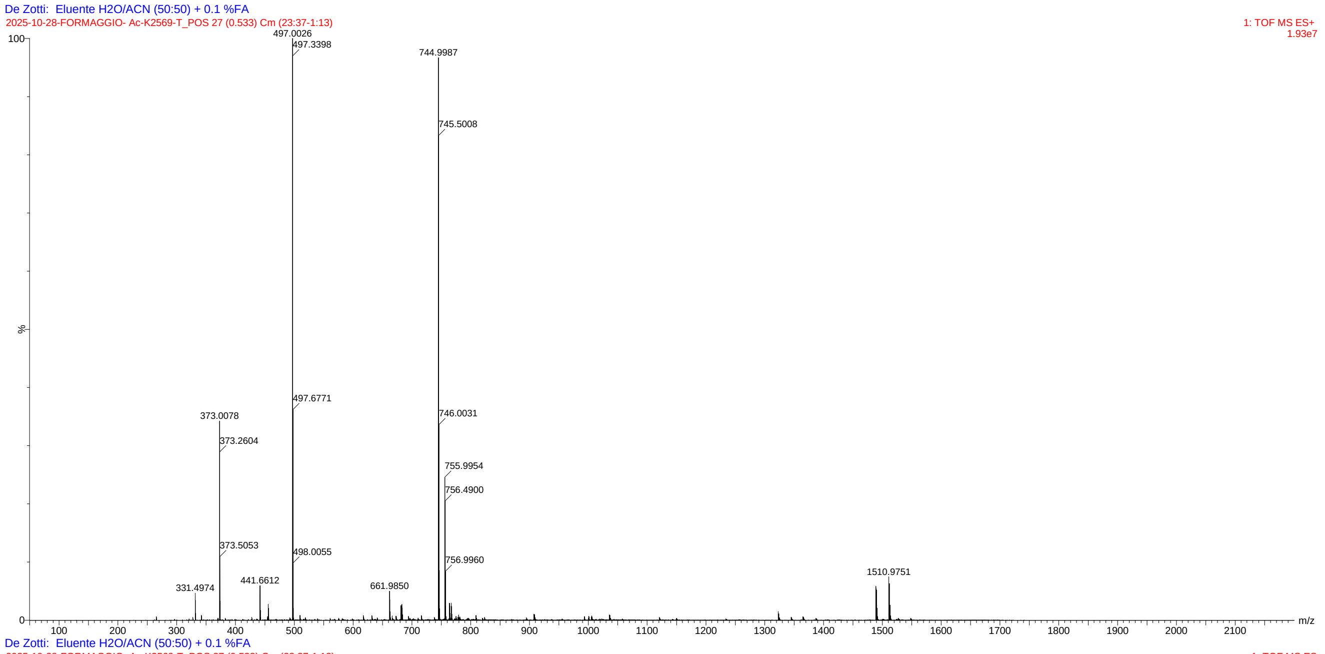

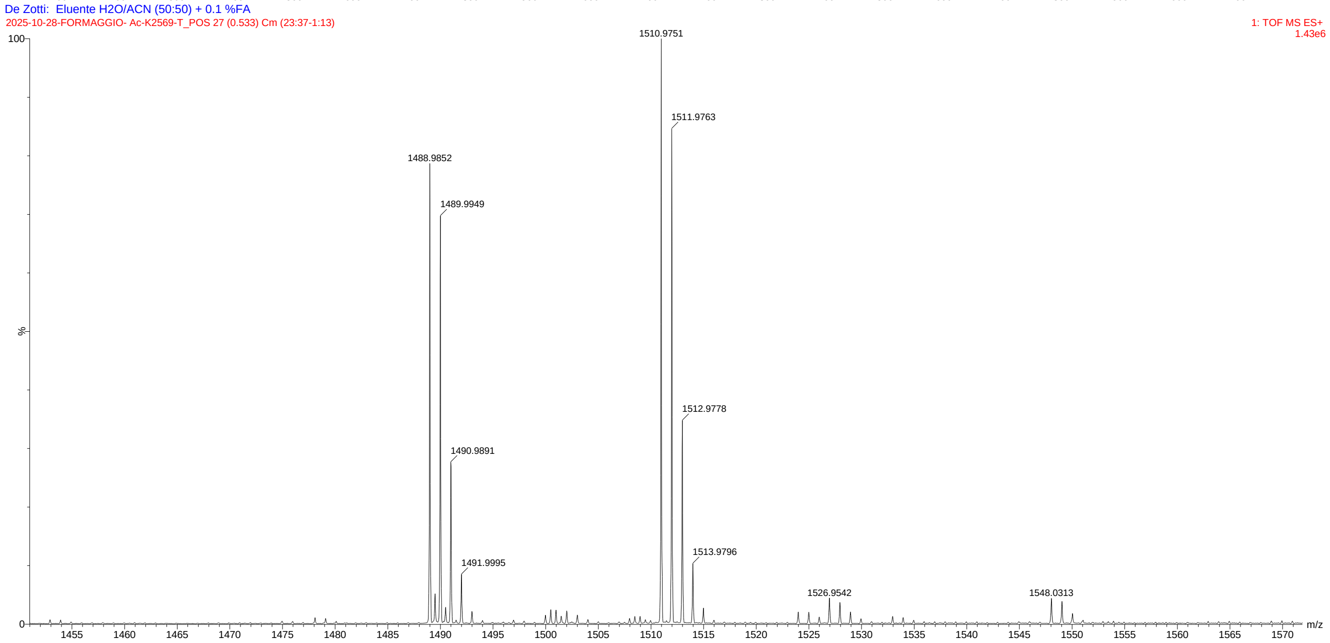

Figure S13. (top) Overall and (bottom) Zoomed ESI-MS spectrum of **Ac-TricK2569-T**. $[M+H]^{+}_{calcd.}$: 1488.9916; $[M+H]^{+}_{found}$: 1488.9852; $[M+Na]^{+}_{found}$: 1510.9751; $[M+K]^{+}_{found}$:1526.9542. $[M+2H]^{2+}_{calcd.}$: 744.9958; $[M+2H]^{2+}_{found}$: 744.9987. $[M+3H]^{3+}_{calcd.}$: 496.9972; $[M+3H]^{3+}_{found}$: 497.0026. $[M+4H]^{4+}_{calcd.}$: 372.9979; $[M+4H]^{4+}_{found}$: 373.0078. Known impurity (4%): Ac-TricK2569-H (reagent, MW: 1321.9536) $[M+4H]^{4+}_{calcd.}$: 331.4884; $[M+4H]^{4+}_{found}$: 331.4808; $[M+3H]^{3+}_{found}$: 441.6612; $[M+2H]^{2+}_{found}$: 661.9850.

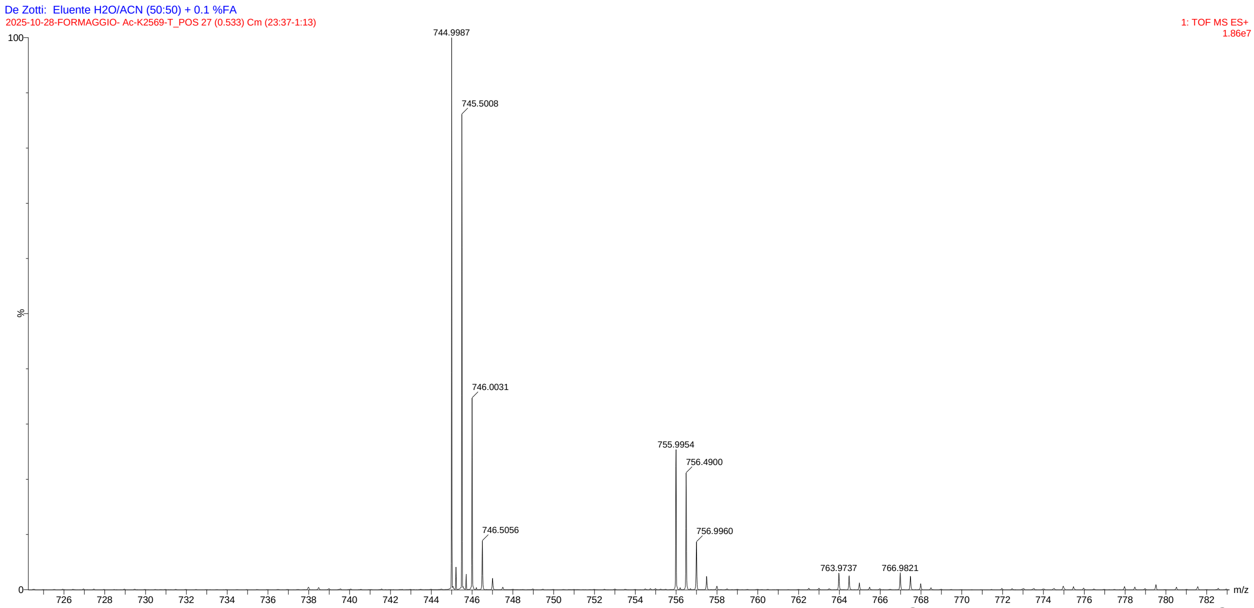

Figure S14. Detail of the ESI-MS spectrum of **Ac-TricK2569-T**. $[M+2H]^{2+}_{calcd.}$: 744.9958; $[M+2H]^{2+}_{found}$: 744.9987. $[M+H+Na]^{2+}_{calcd.}$: 755.9958; $[M+H+Na]^{2+}_{found}$: 755.9954. $[M+2Na]^{2+}_{calcd.}$: 766.9958; $[M+2Na]^{2+}_{found}$: 766.9821.

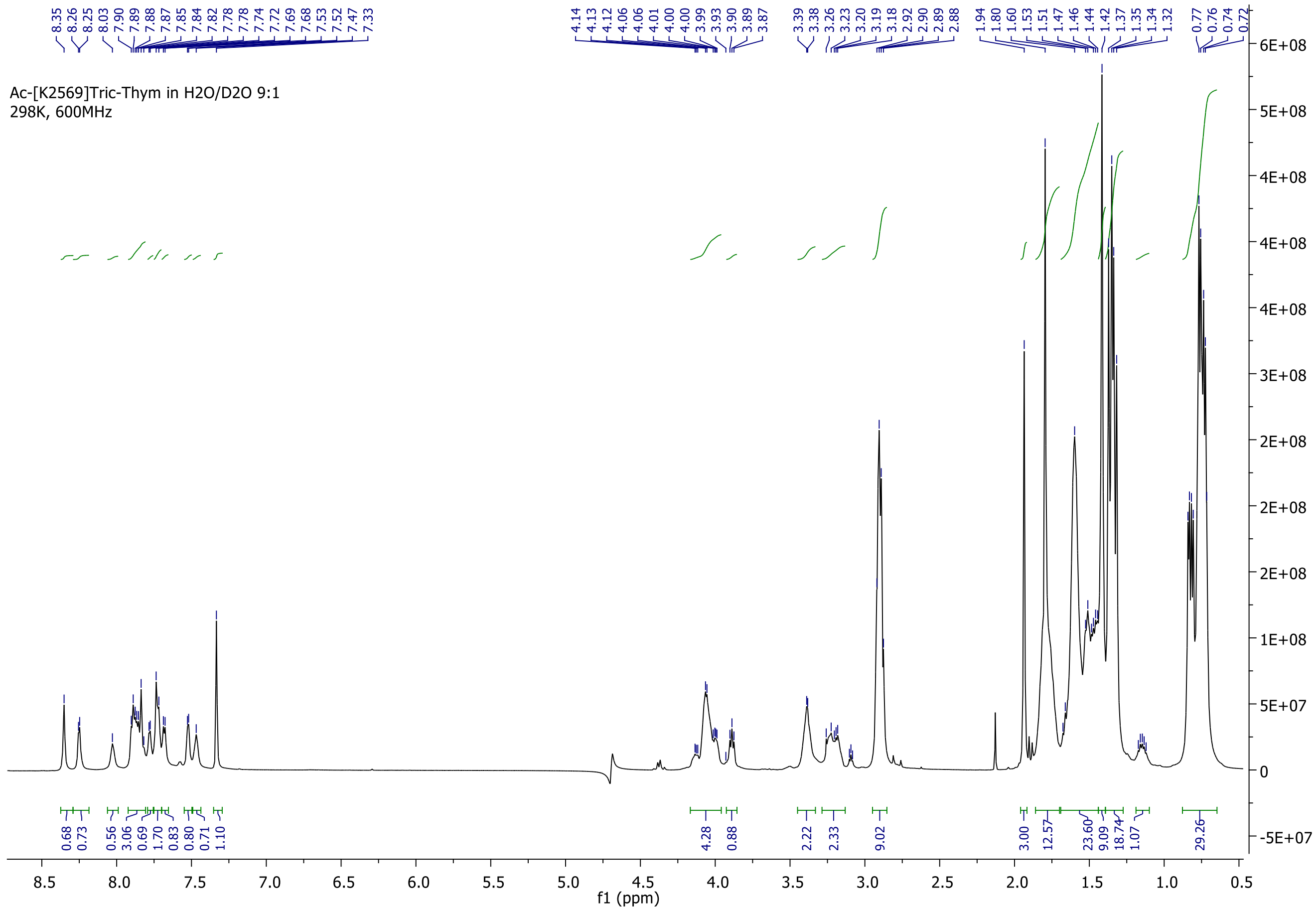

Figure S15. 1D ^1H NMR spectrum of **Ac-TricK2569-T** in H_2O/D_2O 9:1 (600 MHz, 298K). Peptide concentration: 2mM.

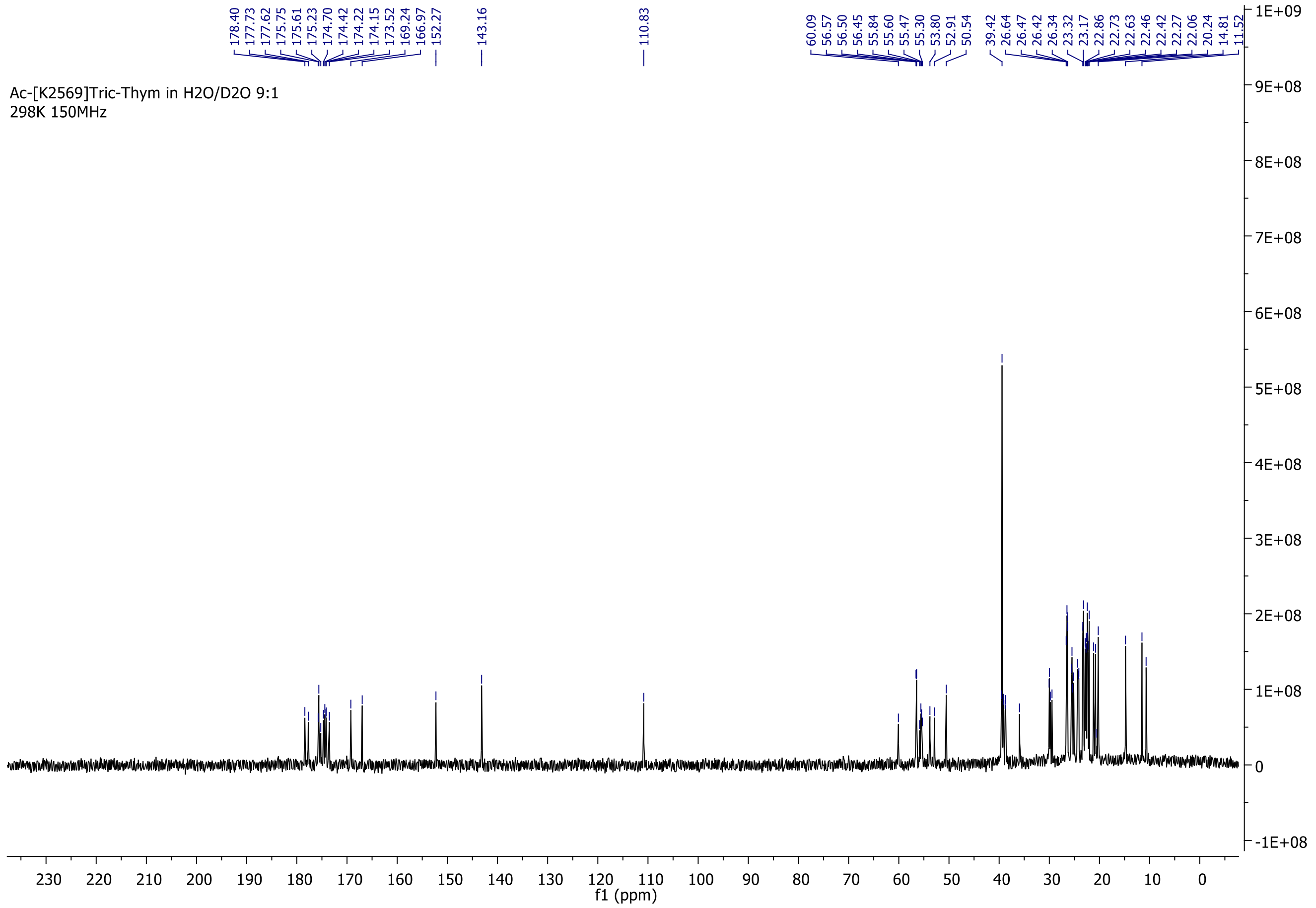

Figure S16. 1D ^{13}C NMR spectrum of **Ac-TricK2569-T** in H_2O/D_2O 9:1 (150 MHz, 298K). Peptide concentration: 2mM.

Dynamic Light Scattering (DLS) spectra. DLS analysis (scattering angle: 173°) was performed on all samples at different peptide/lipid (P/L) ratios. The presence of a Thymine at the C-terminus stabilizes the peptide-induced aggregates even at a peptide concentration above the neutralization, when aggregation is reversible, this is an indication that fusion is probably occurring. The DLS analysis on the fused SUVs are not of a good quality due to their huge dimensions (diameter >10'000nm). Volume distributions derived from dynamic light scattering measurements have been used not as absolute but to compare the samples and estimate the relative amounts of the multiple size peaks.

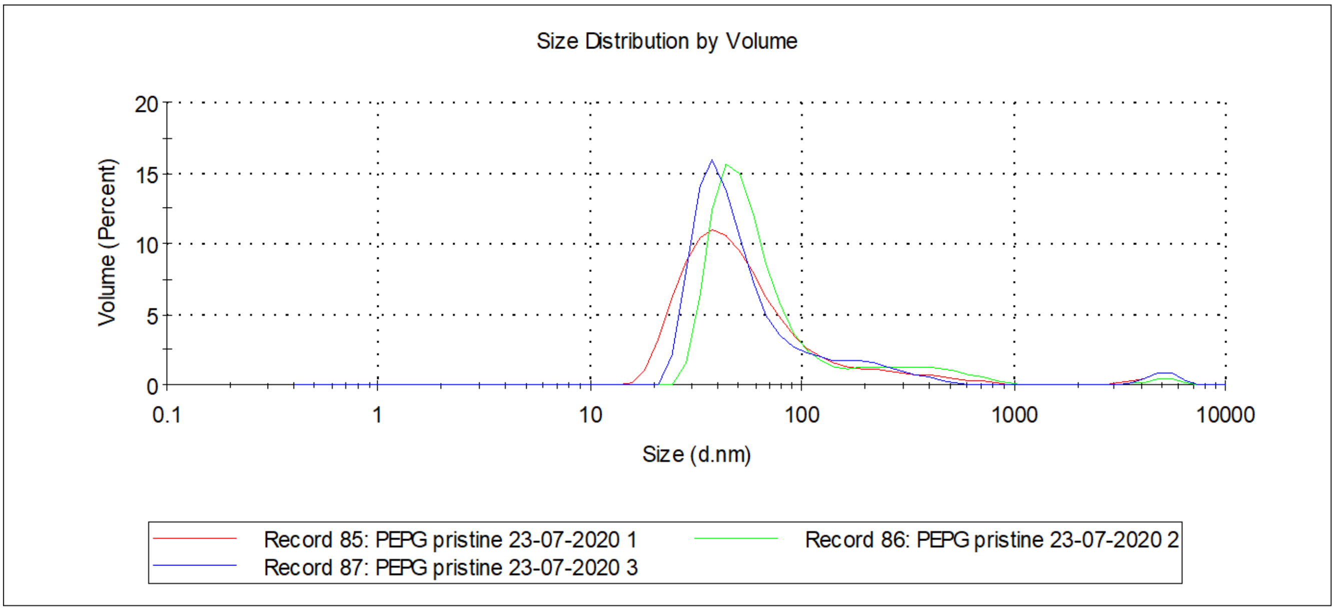

Figure S17. Size graph by volume on one of the several samples of pristine PEPG SUV analyzed. Z-Average diameter: 112.5±0.6nm.

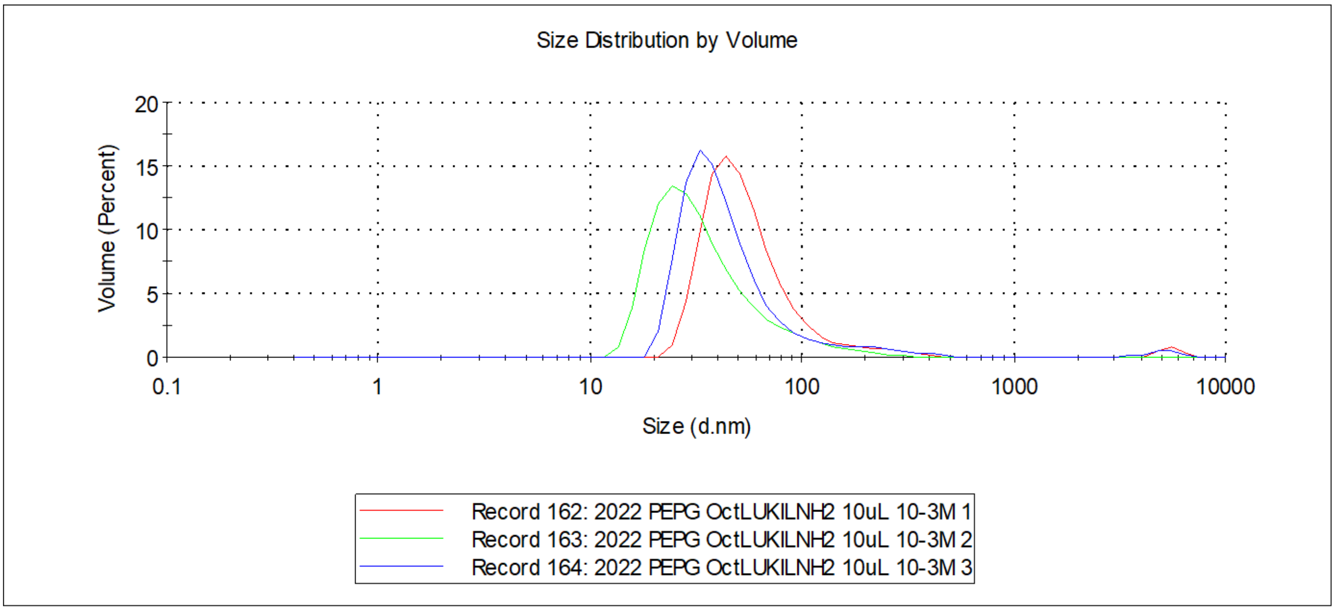

Figure S18. Size graph by volume obtained for a reference, short peptide that does not promote aggregation, on PEPG SUV. Peptide/lipid ratio (P/L) 1:12 [corresponding to the addition of 10μL 10^{-3}M]. Z-Average diameter: 96.2±7.6nm.

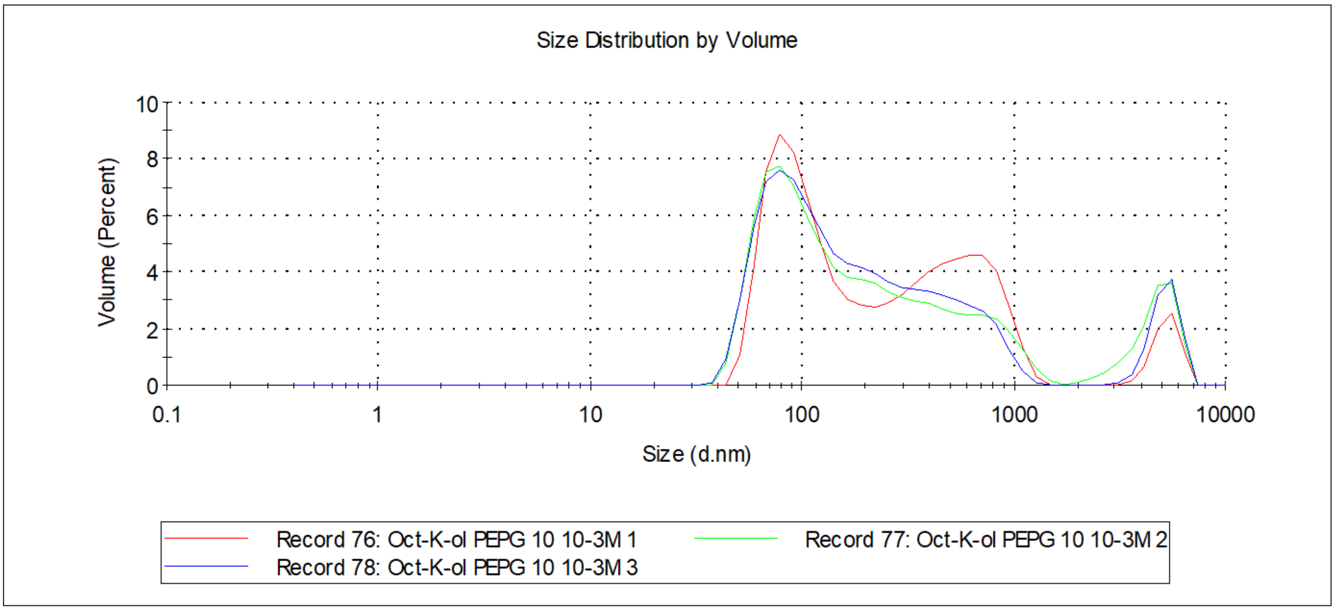

Figure S19. Size graph by volume for **Oct-TricK2569-Lol** at a peptide/lipid ratio (P/L) 1:12 [corresponding to the addition of 10µL 10^{-3}M], on PEPG SUV. Z-Average diameter: 195.6±2.7nm.

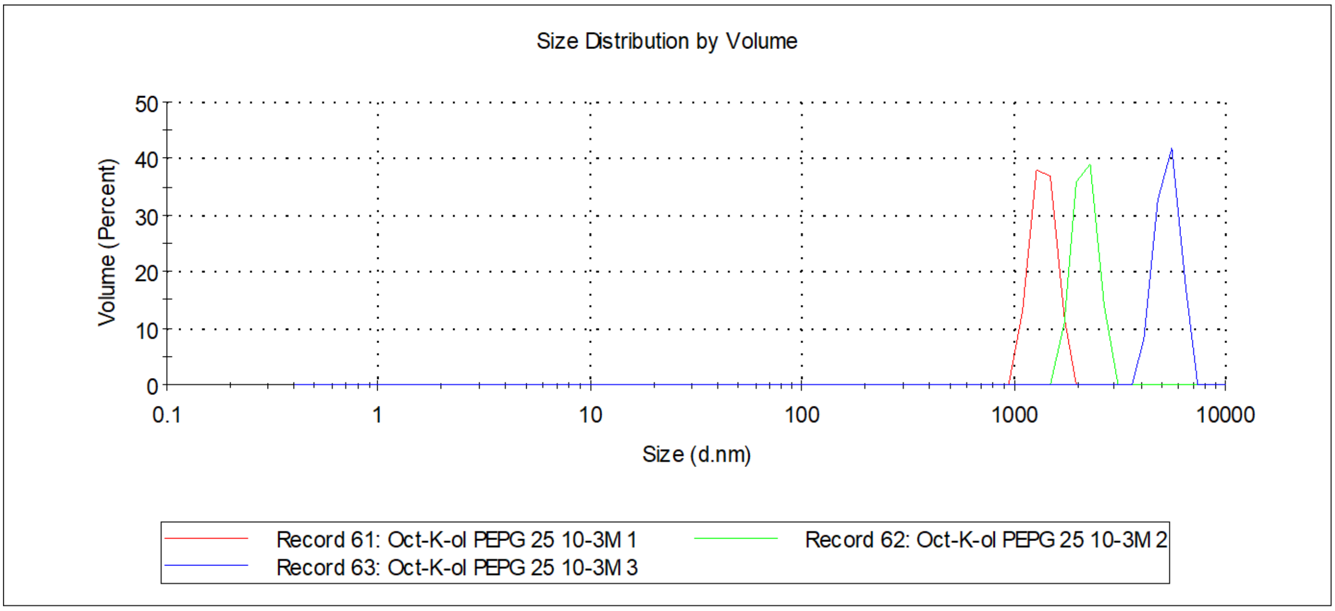

Figure S20. Size graph by volume for Oct-TricK2569-Lol at P/L 1:6 [corresponding to the addition of 25µL 10^{-3}M], on PEPG SUV. Z-Average diameter: 5822±1880nm.

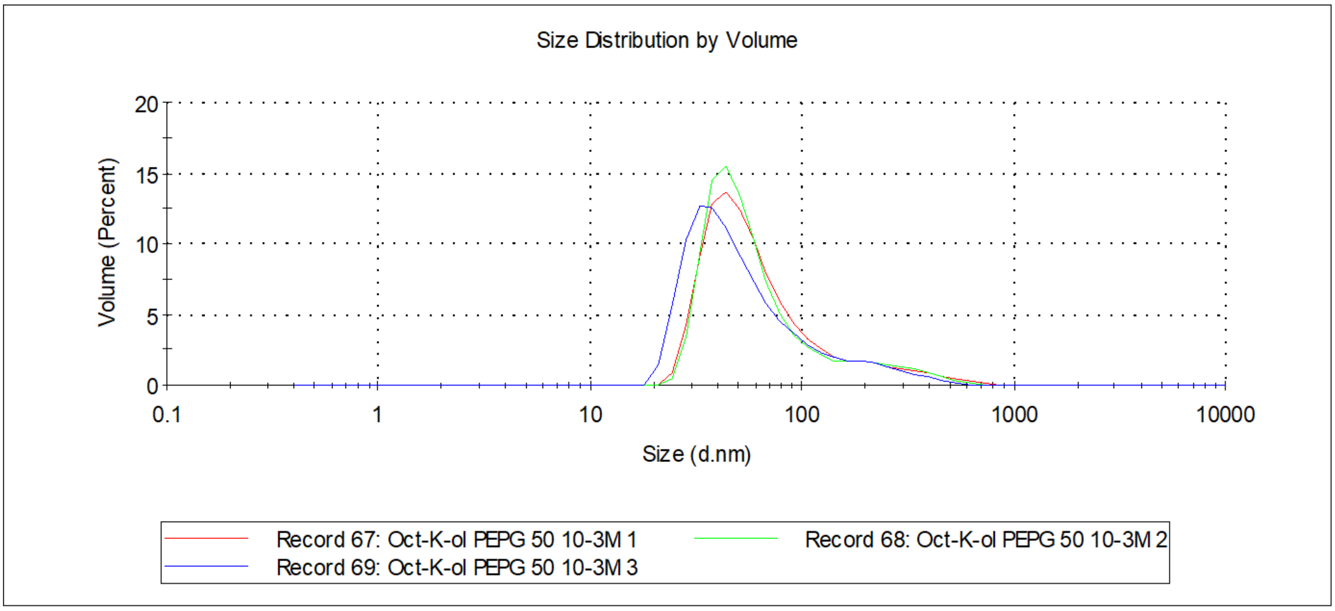

Figure S21. Size graph by volume for Oct-TricK2569-Lol at P/L 1:3 [corresponding to the addition of 50µL 10^{-3}M], on PEPG SUV. Z-Average diameter: 106.9±0.1nm.

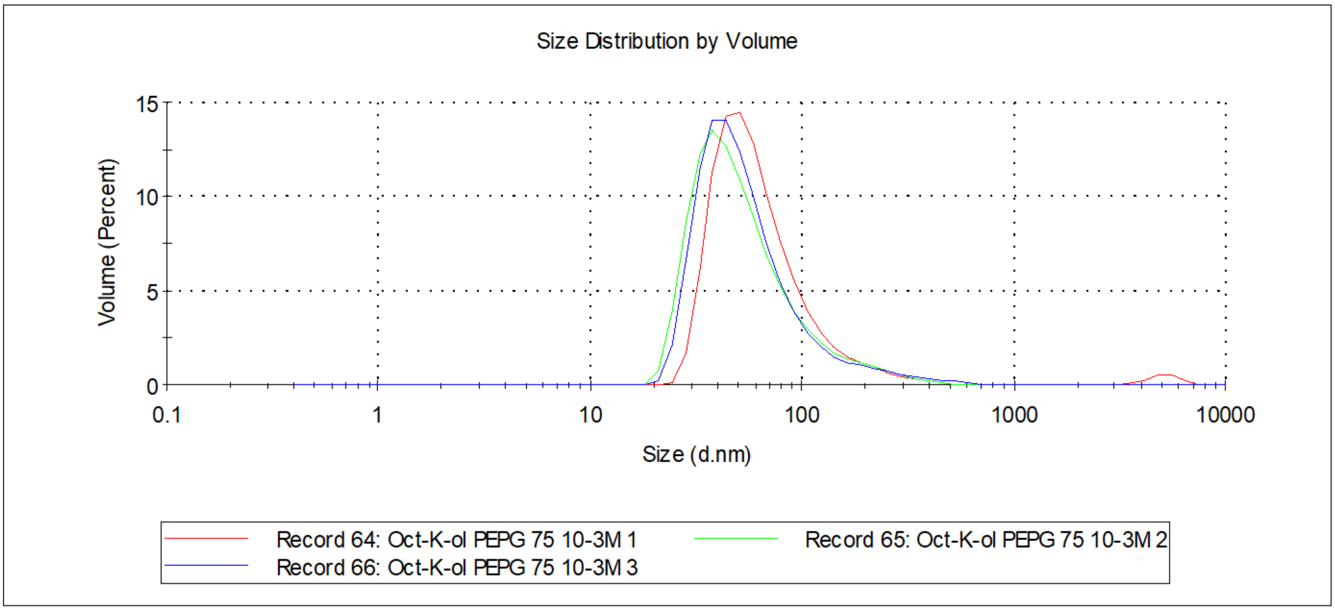

Figure S22. Size graph by volume for Oct-TricK2569-Lol at P/L 1:2 [corresponding to the addition of 75µL 10^{-3}M], on PEPG SUV. Z-Average diameter: 92.7±1.4nm.

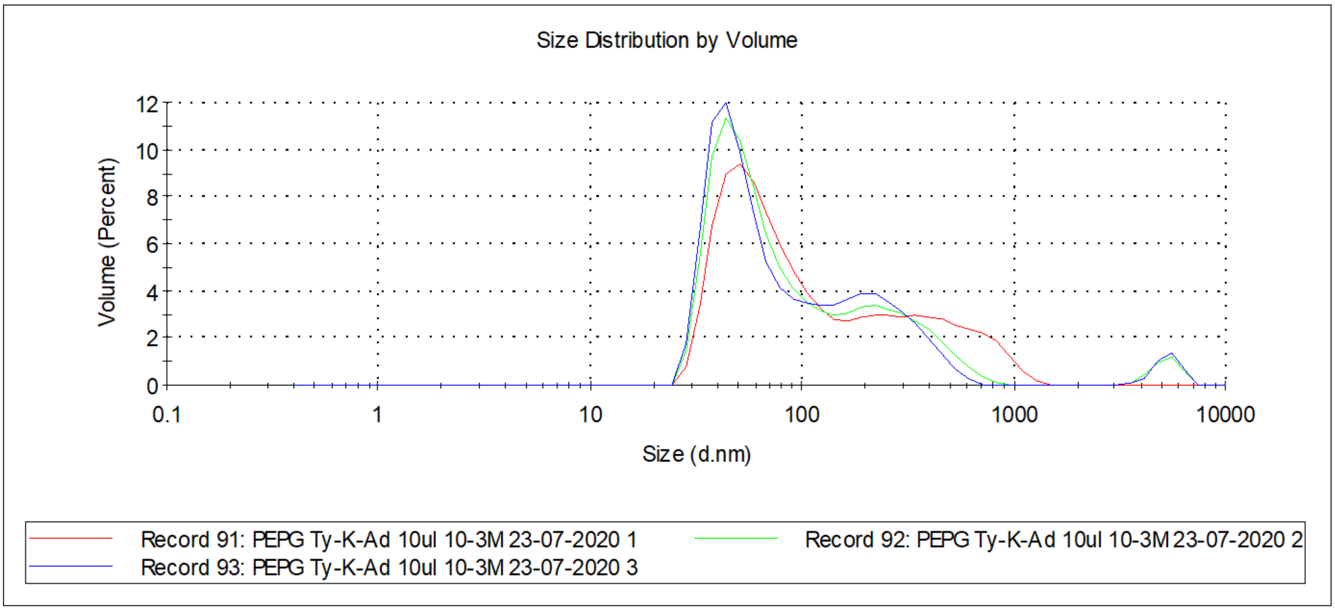

Figure S23. Size graph by volume for **T-TricK2569-A** at a peptide/lipid ratio (P/L) 1:12 [corresponding to the addition of 10μL 10^{-3}M], on PEPG SUV. Z-Average diameter: 157.3±4.1nm.

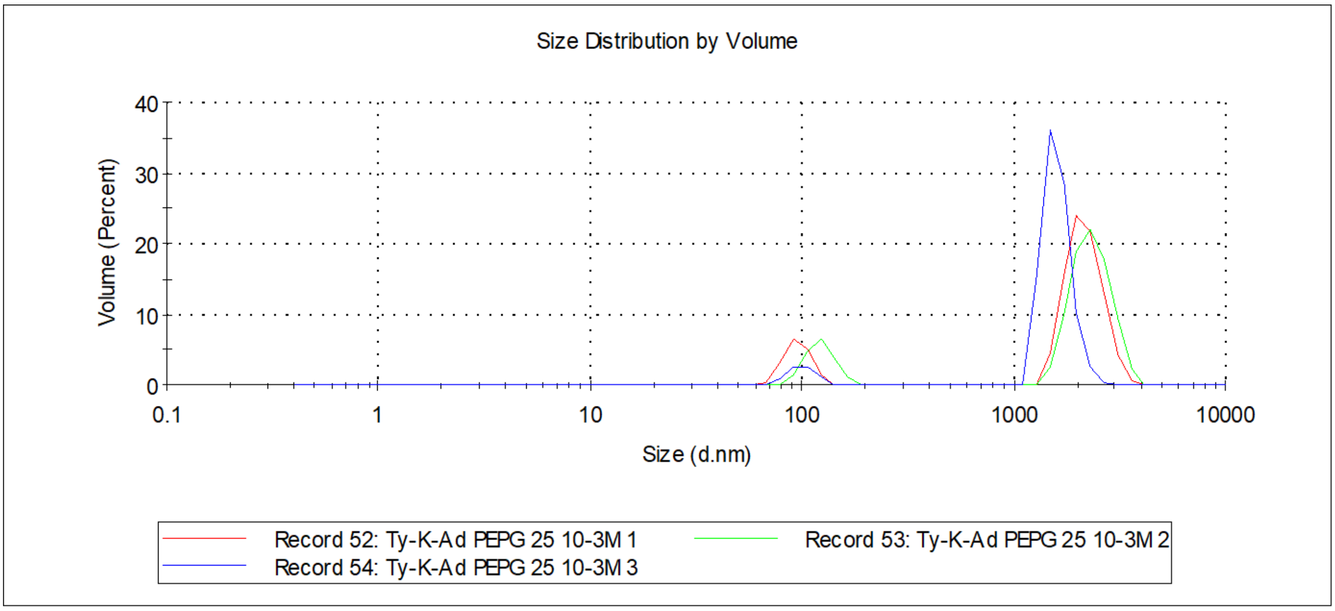

Figure S24. Size graph by volume for **T-TricK2569-A** at P/L 1:6 [corresponding to the addition of 25μL 10^{-3}M], on PEPG SUV. Z-Average diameter: 3191±173nm.

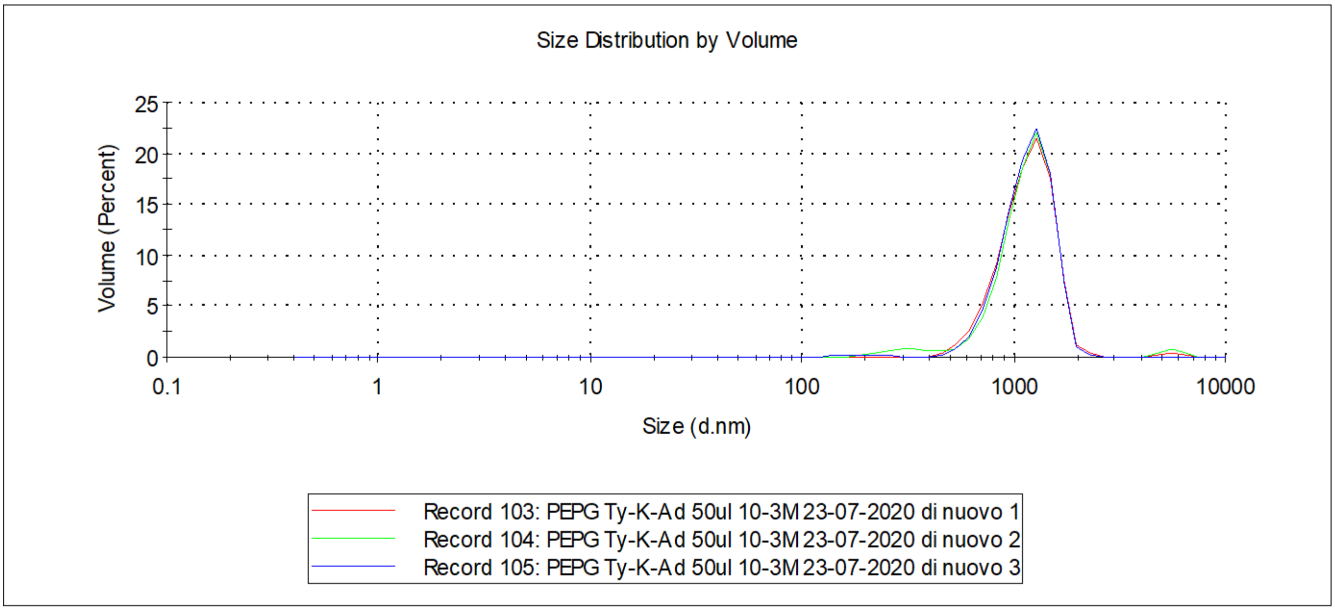

Figure S25. Size graph by volume for **T-TricK2569-A** at P/L 1:3 [corresponding to the addition of 50μL 10^{-3}M], on PEPG SUV. Z-Average diameter: 948±25nm.

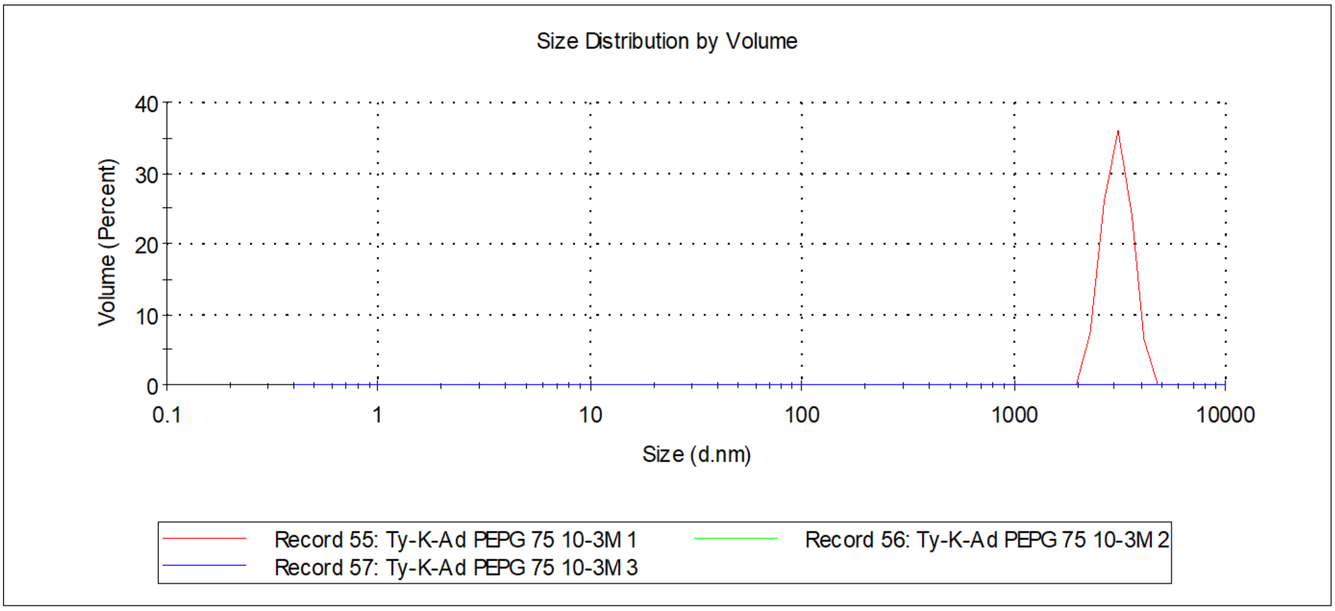

Figure S26. Size graph by volume for **T-TricK2569-A** at P/L 1:2 [corresponding to the addition of 75μL 10^{-3}M], on PEPG SUV. Z-Average diameter: 10530±8604nm. Both the second and third repetitions were out of range (>10'000nm).

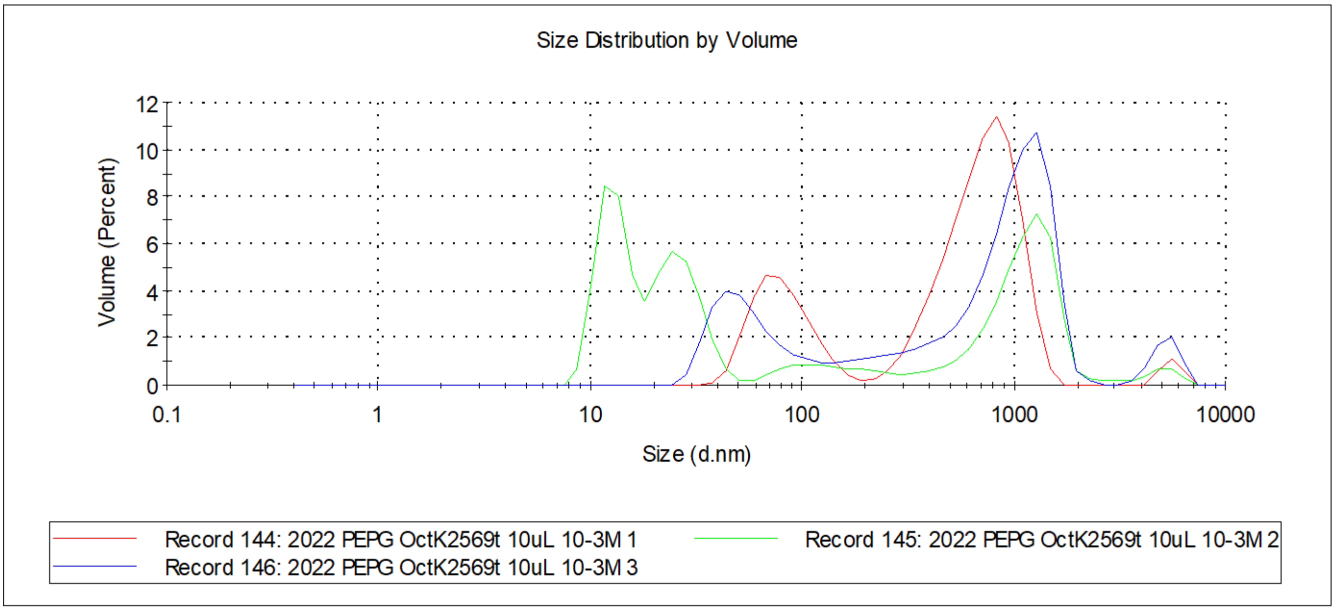

Figure S27. Size graph by volume for **Oct-TricK2569-T** at a peptide/lipid ratio (P/L) 1:12 [corresponding to the addition of 10µL 10^{-3}M], on PEPG SUV. Z-Average diameter: 327±17nm.

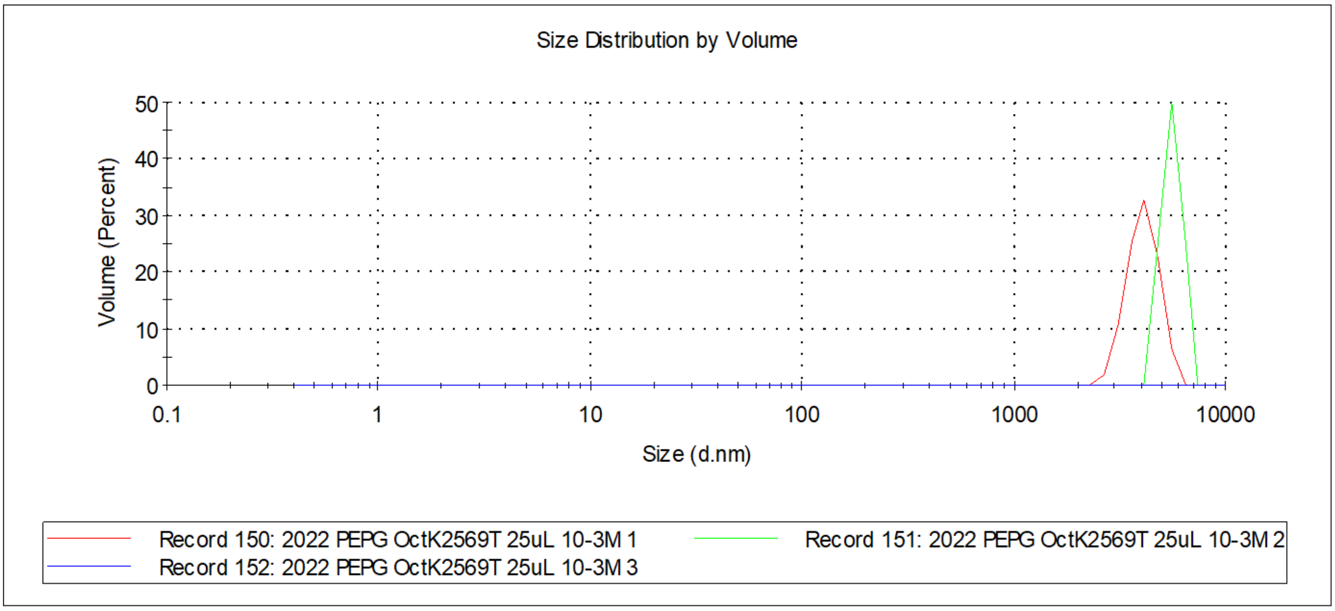

Figure S28. Size graph by volume for Oct-TricK2569-T at P/L 1:6 [corresponding to the addition of 25µL 10^{-3}M], on PEPG SUV. Z-Average diameter over the two in-range repetitions: 4971±1887nm. Third repetition was out of range (diameter > 10'000 nm).

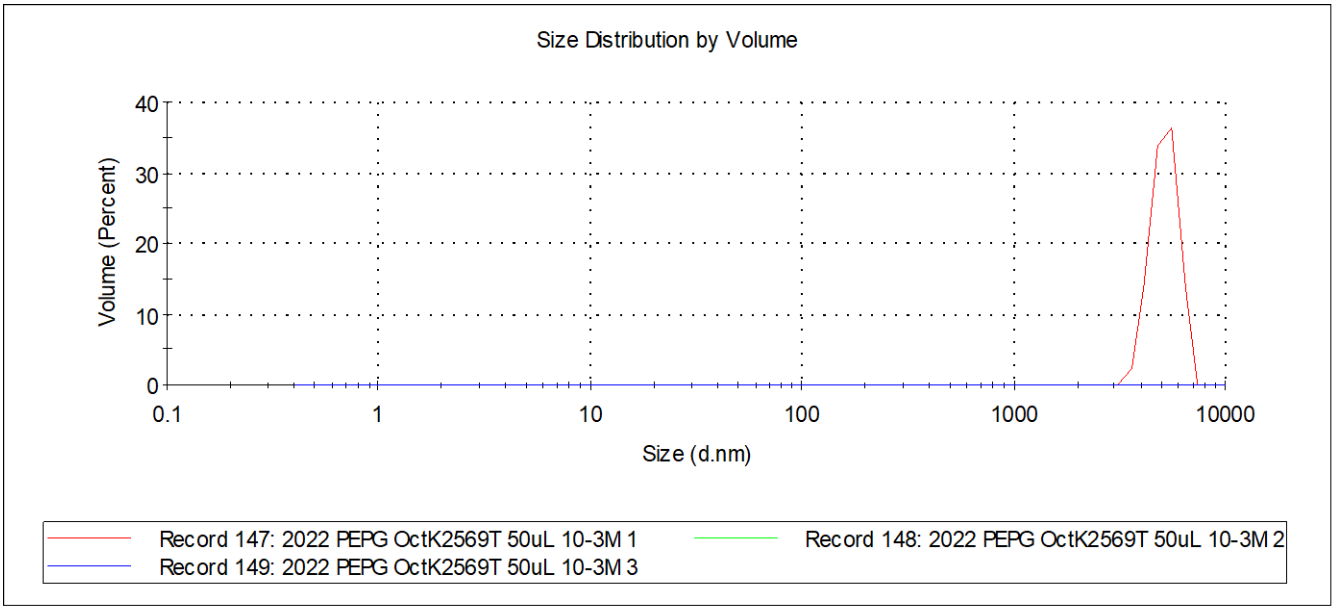

Figure S29. Size graph by volume for Oct-TricK2569-T at P/L 1:3 [corresponding to the addition of 50μL 10^{-3}M], on PEPG SUV. Z-Average diameter: 18750±16570 nm. Both the second and third repetitions were out of range.

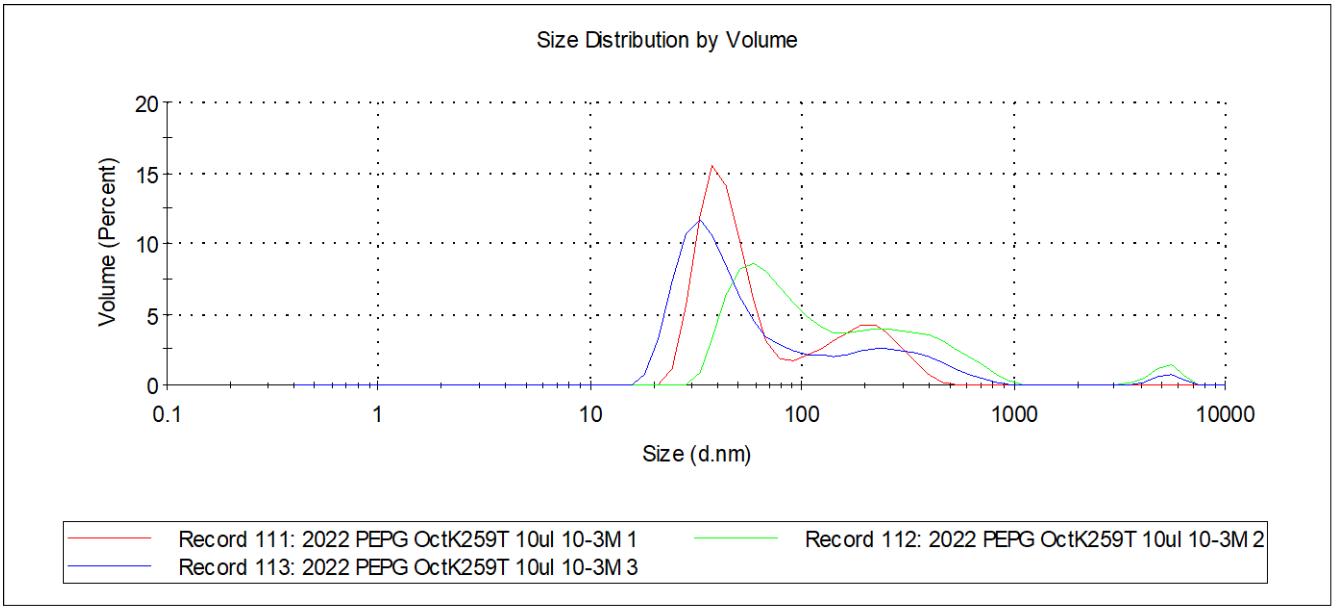

Figure S30. Size graph by volume for **Oct-TricK259-T** at a peptide/lipid ratio (P/L) 1:12 [corresponding to the addition of 10µL 10^{-3}M], on PEPG SUV. Z-Average diameter: 162.4±7.8nm.

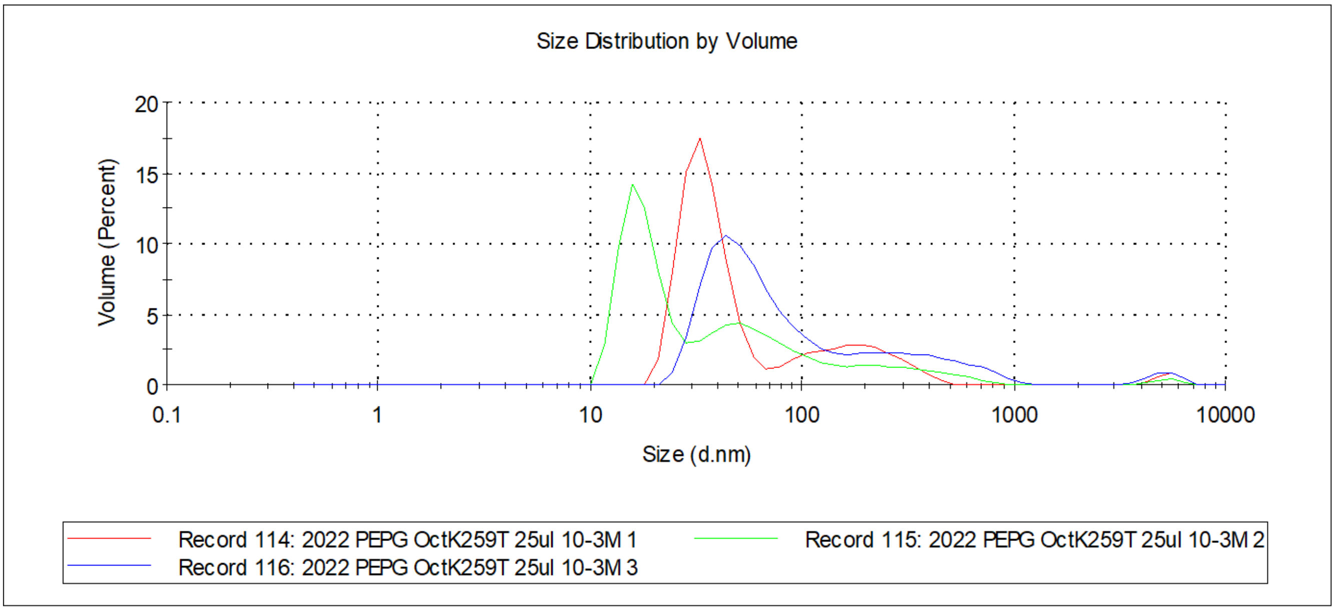

Figure S31. Size graph by volume for Oct-TricK259-T at P/L 1:6 [corresponding to the addition of 25µL 10^{-3}M], on PEPG SUV. Z-Average diameter: 147.3±6.6nm.

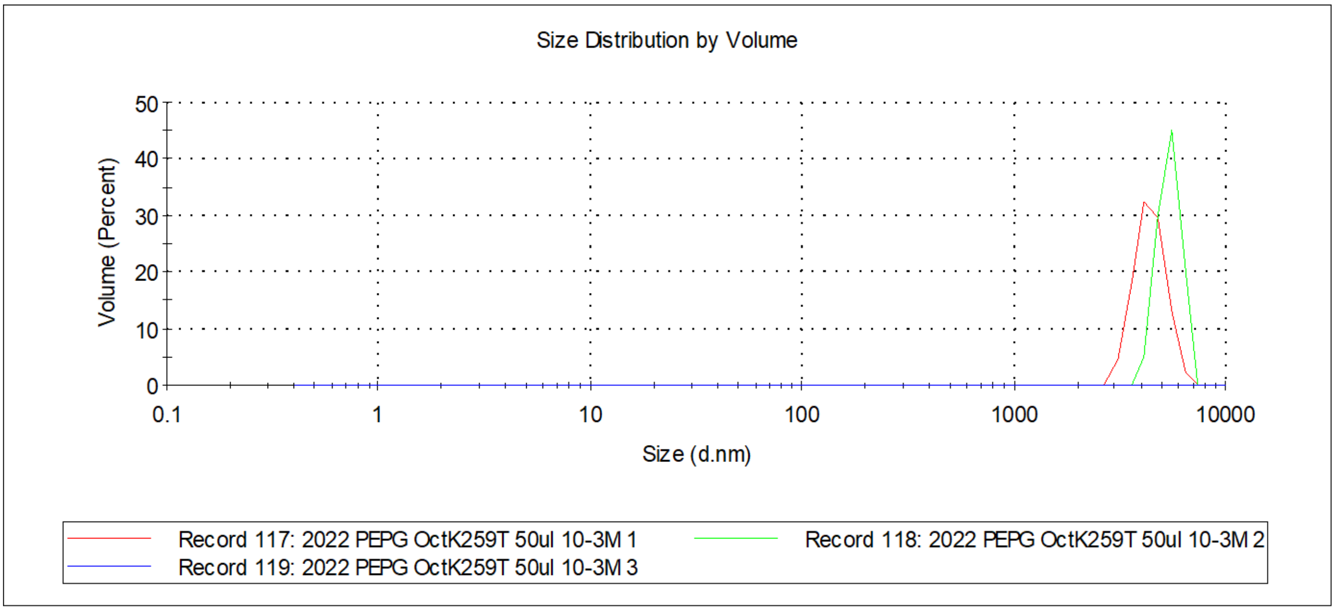

Figure S32. Size graph by volume for Oct-TricK259-T at P/L 1:3 [corresponding to the addition of 50μL 10^{-3}M], on PEPG SUV. Z-Average diameter: 7830±3466nm. Third repetition was out of range.

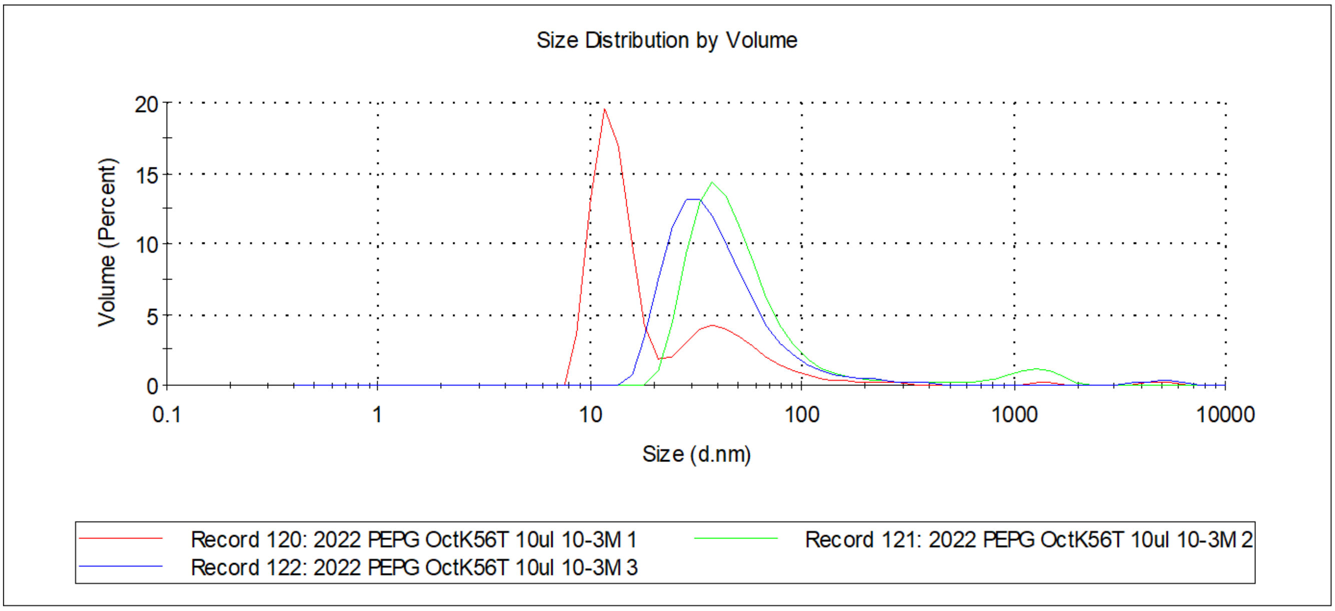

Figure S33. Size graph by volume for **Oct-TricK56-T** at a peptide/lipid ratio (P/L) 1:12 [corresponding to the addition of 10µL 10^{-3}M], on PEPG SUV. Z-Average diameter: 78.1±1.5nm.

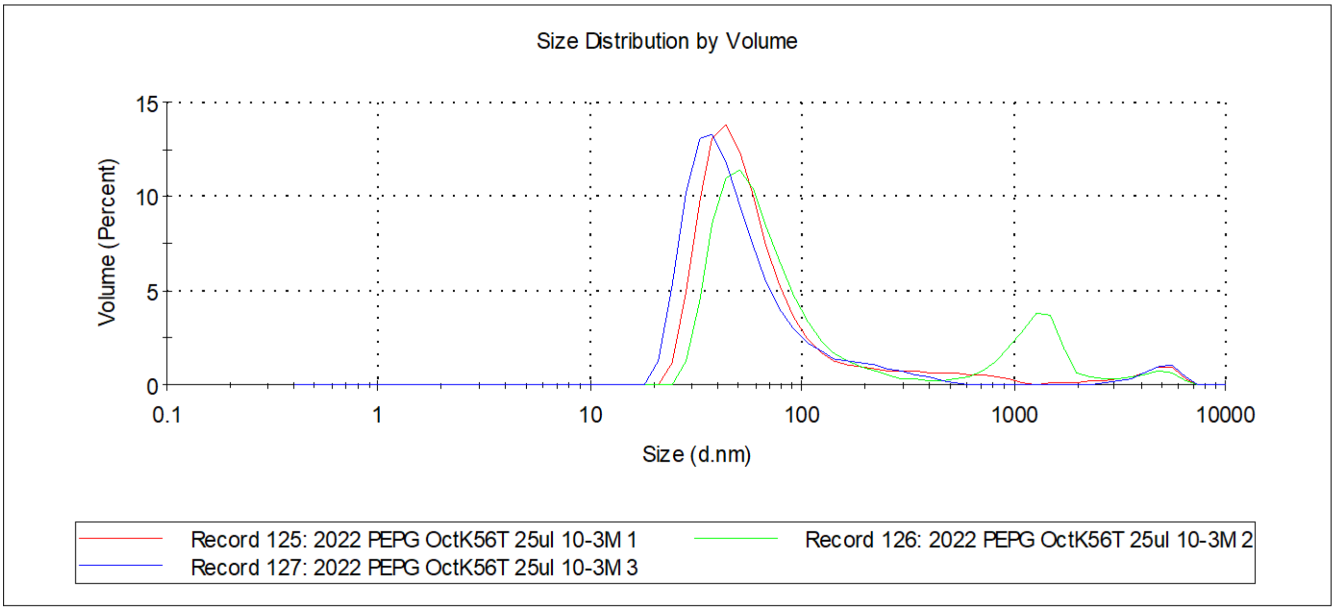

Figure S34. Size graph by volume for Oct-TricK56-T at P/L 1:6 [corresponding to the addition of 25µL 10^{-3}M], on PEPG SUV. Z-Average diameter: 107.8±2.1nm.

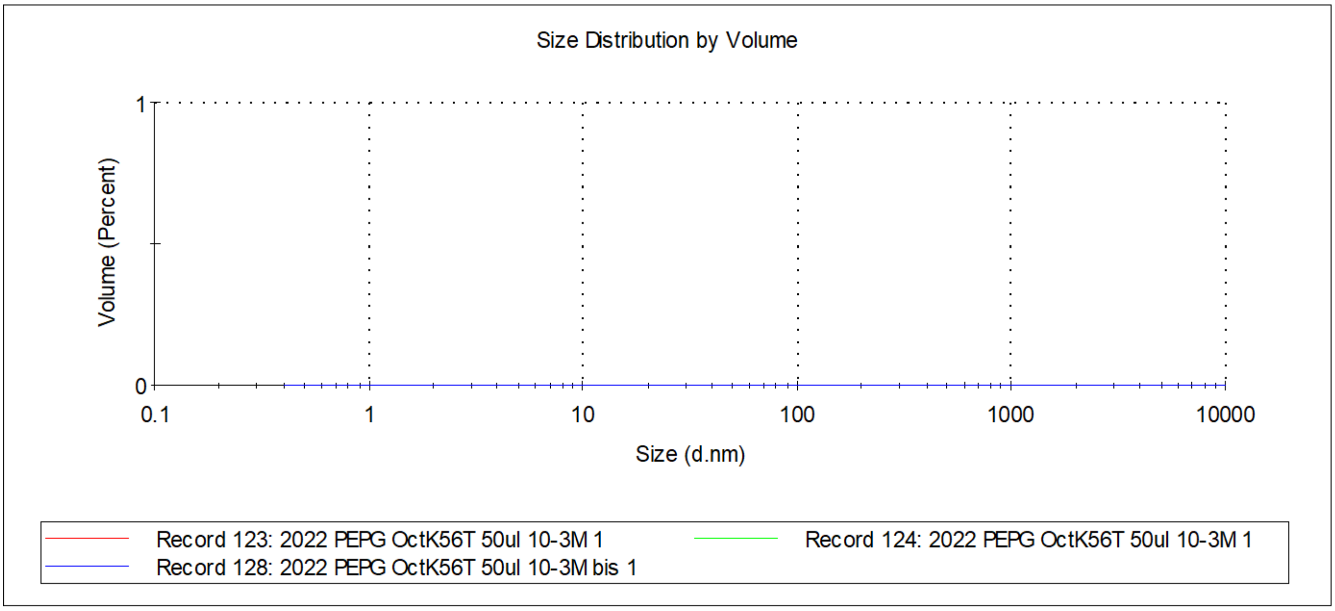

Figure S35. Size graph by volume for Oct-TricK56-T at P/L 1:3 [corresponding to the addition of 50µL 10^{-3}M], on PEPG SUV. Z-Average diameter: 28920±15460nm. All repetitions were out of range (>10'000nm).

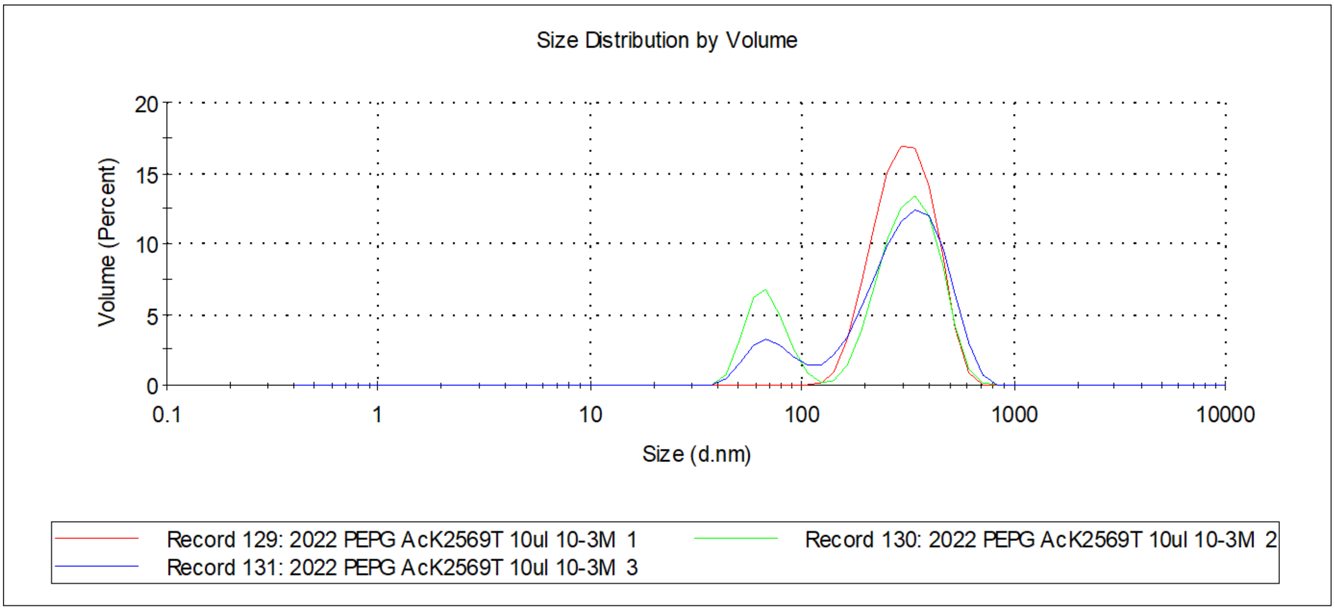

Figure S36. Size graph by volume for **Ac-TricK2569-T** at a peptide/lipid ratio (P/L) 1:12 [corresponding to the addition of 10µL 10^{-3}M], on PEPG SUV. Z-Average diameter: 316.4±8.7nm.

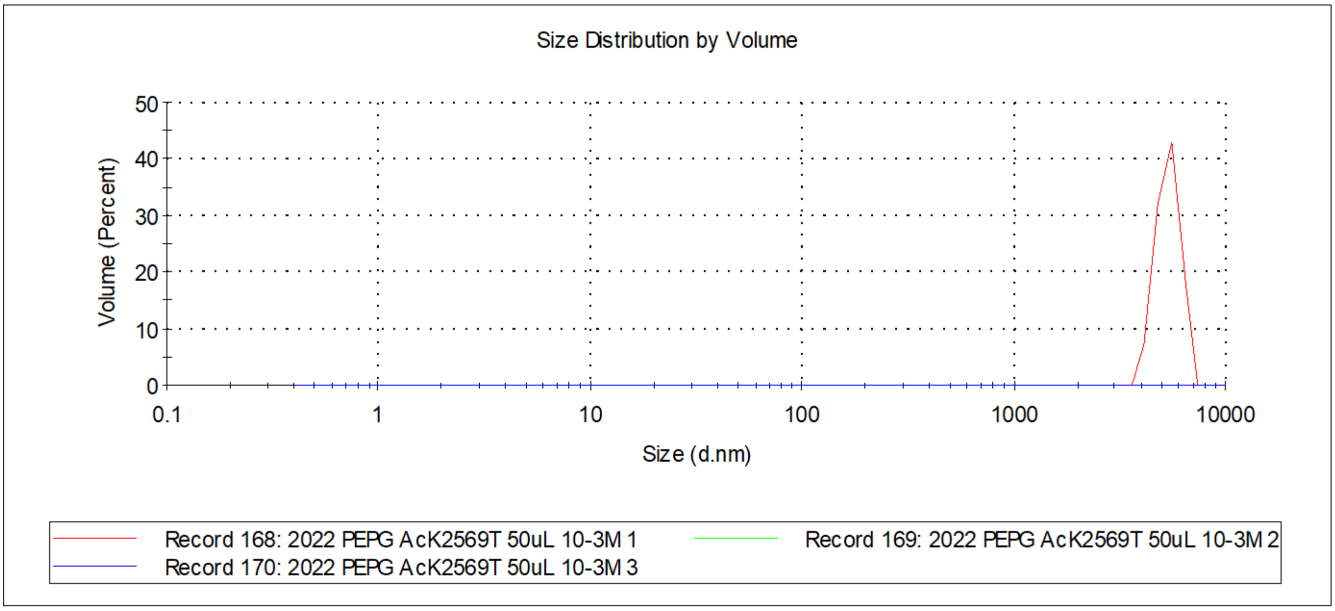

Figure S37. Size graph by volume for Ac-TricK2569-T at P/L 1:3 [corresponding to the addition of 50μL 10^{-3}M], on PEPG SUV. Z-Average diameter: 13200±5247nm. Both the second and third repetitions were out of range (>10'000nm).

Isothermal Titration Calorimetry (ITC). ITC analysis was performed on the peptides: Oct-K2569Tric-Lol, Oct-K2569Tric-T, and T-K2569Tric-A as a function of increasing peptide/lipid (P/L) ratios. SUV lipid composition: POPE/POPG 7:3. SUV concentration in the Cell: 0.1 mM. Peptide concentration in the syringe: 33.3 mM. Both SUVs and peptides were dissolved in the same buffer as leakage experiments, namely Hepes buffer (5 mm Hepes, 100 mM NaCl, pH 7.4). The data confirm the results obtained by DLS, with the presence of a Thymine at the C-terminus stabilizing the Enthalpy of the process. In contrast, Oct-K2569Tric-Lol induced aggregation shows a change in the Enthalpy curve, corresponding at the P/L ratio when disaggregation should occur. All analyses confirm the endothermic nature of both the aggregation and fusion process.

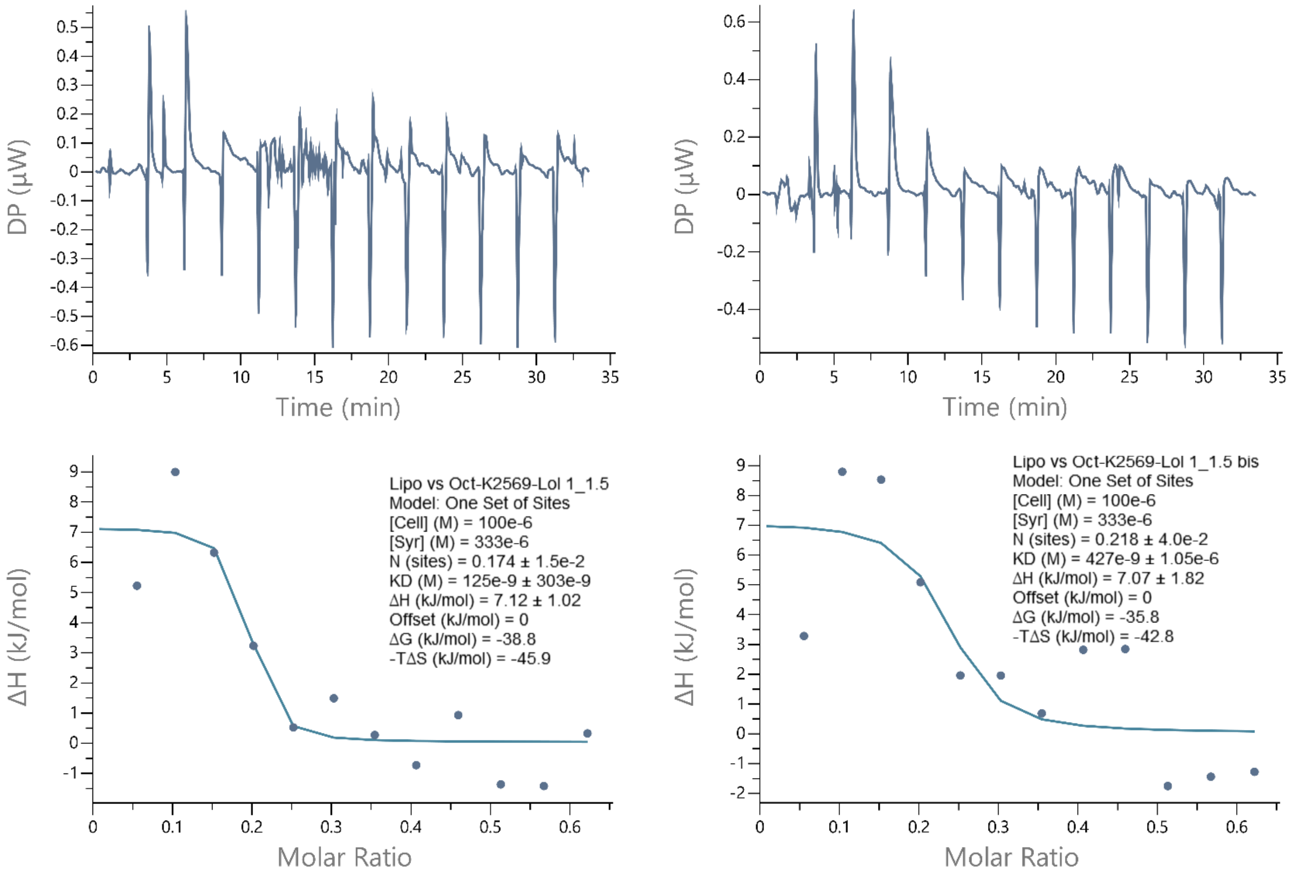

Figure S38. Results of repeated ITC analysis of SUVs of POPE/POPG 7:3 titrated with peptide Oct-K2569Tric-Lol performed at 20°C (reference power setting applied: 5 and 10 µcal/sec, respectively). Raw trace (top) and integrated isotherm (bottom) of the calorimetric titration.

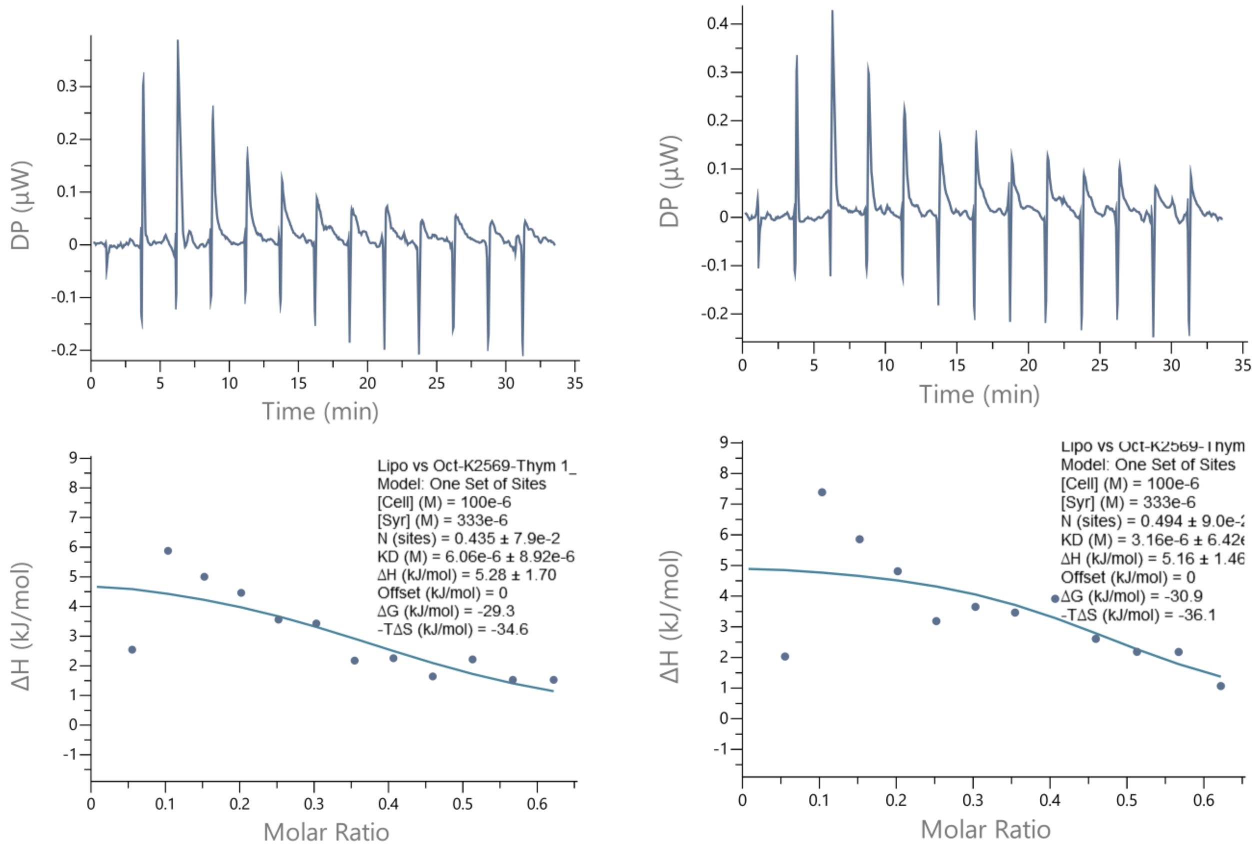

Figure S39. Results of repeated ITC analysis of SUVs of POPE/POPG 7:3 titrated with peptide Oct-K2569Tric-T performed at 20°C (reference power setting applied: 5 and 10 µcal/sec, respectively). Raw trace (top) and integrated isotherm (bottom) of the calorimetric titration.

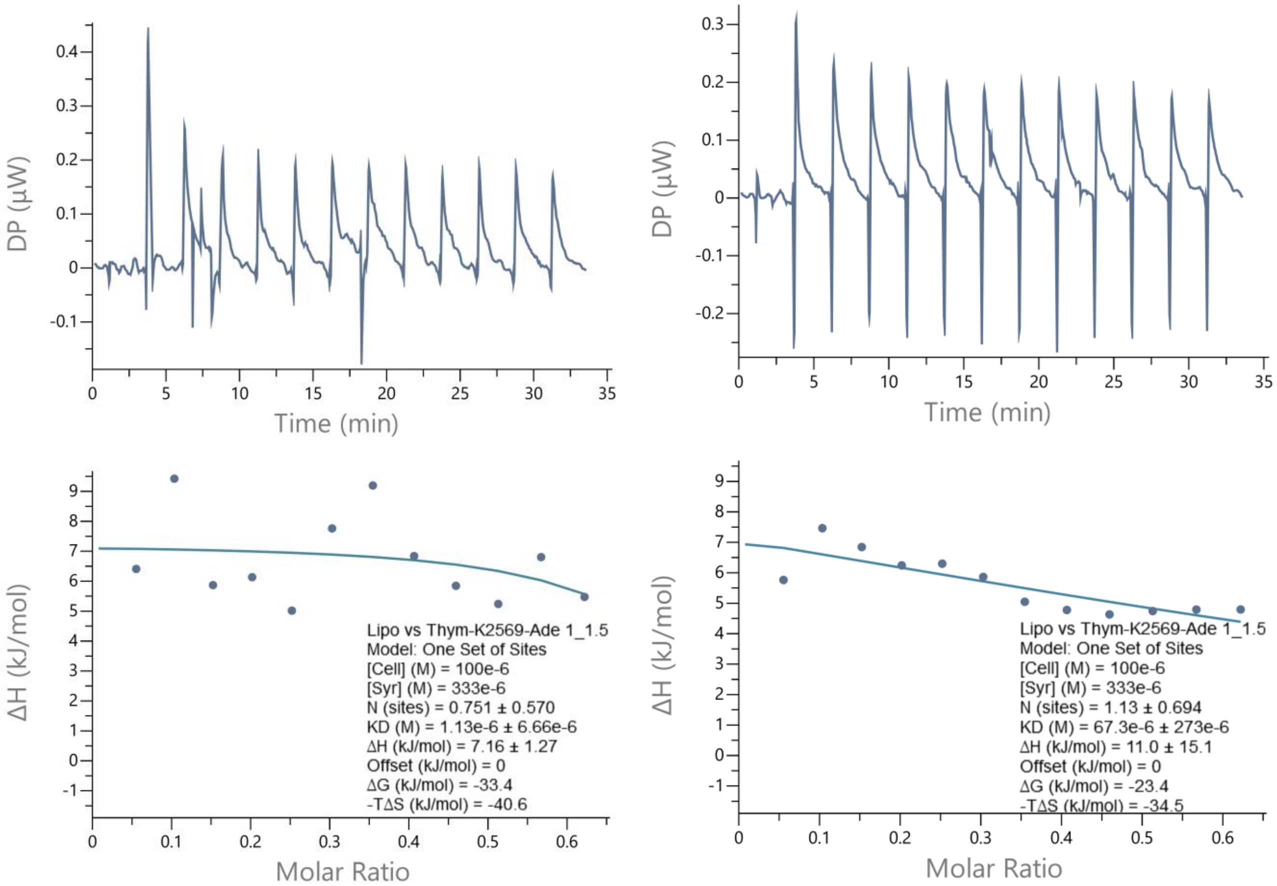

Figure S40. Results of repeated ITC analysis of SUVs of POPE/POPG 7:3 titrated with peptide T-K2569Tric-A performed at 20°C (reference power setting applied: 5 and 10 µcal/sec, respectively). Raw trace (top) and integrated isotherm (bottom) of the calorimetric titration.

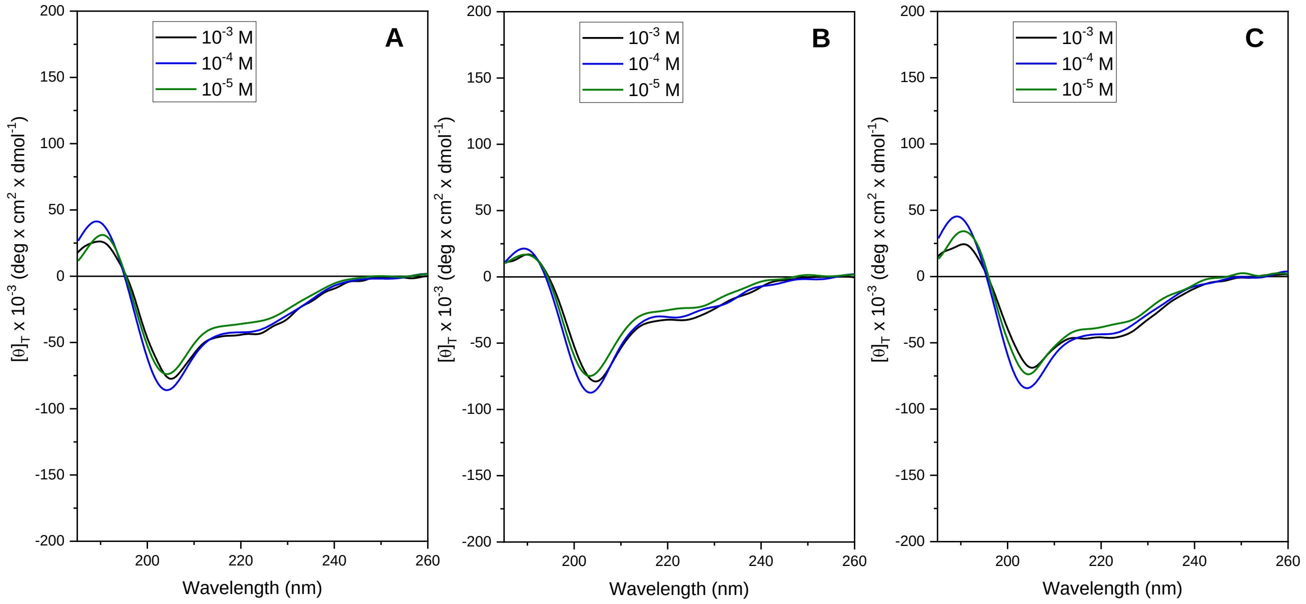

Figure S41. CD analysis as a function of peptide concentration. Dichroic profiles ($[\theta]_T$, total ellipticity) recorded in water for Oct-K2569Tric-T (**A**), Oct-K2569Tric-Lol (**B**), and T-K2569Tric-A (**C**) at 10^{-3} M (black) 10^{-4} (blue) and 10^{-5} M (green).

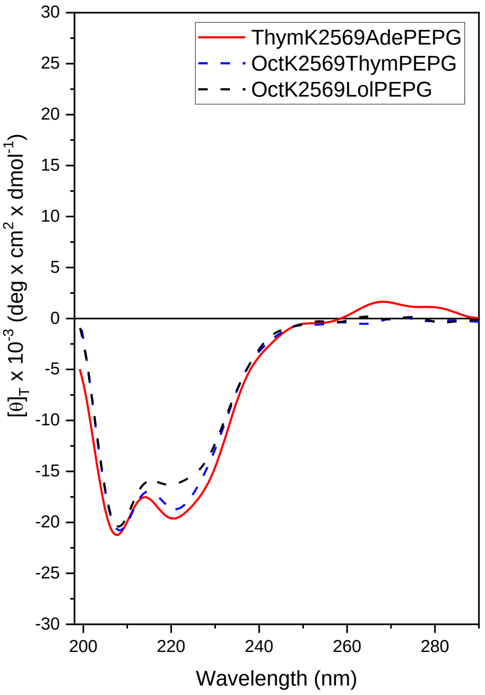

Figure S42. Dichroic profiles ($[\theta]_T$, total ellipticity) recorded in the presence of PEPG 7:3 liposomes for trichogin analogs: Oct-K2569Tric-Lol (dashed line), Oct-K2569Tric-T (blue line), and T-K2569Tric-A (red line). Peptide/Lipid molar ratio: 1/2. The CD profile of peptide T-K2569Tric-A, which bears the two complementary nucleobases Adenine and Thymine, featured a positive signal at about 267-270 nm, corresponding to the UV absorption of the two nucleobases.

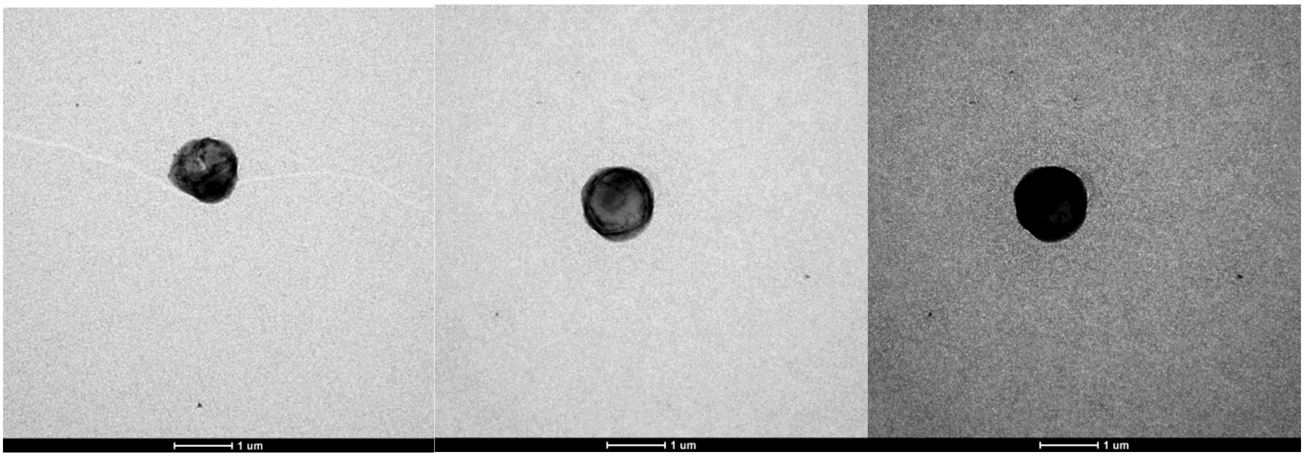

Figure S43. Additional TEM images for PEPG SUVs treated with peptide Thym-K2569-Thym at a P/L = 1/3.

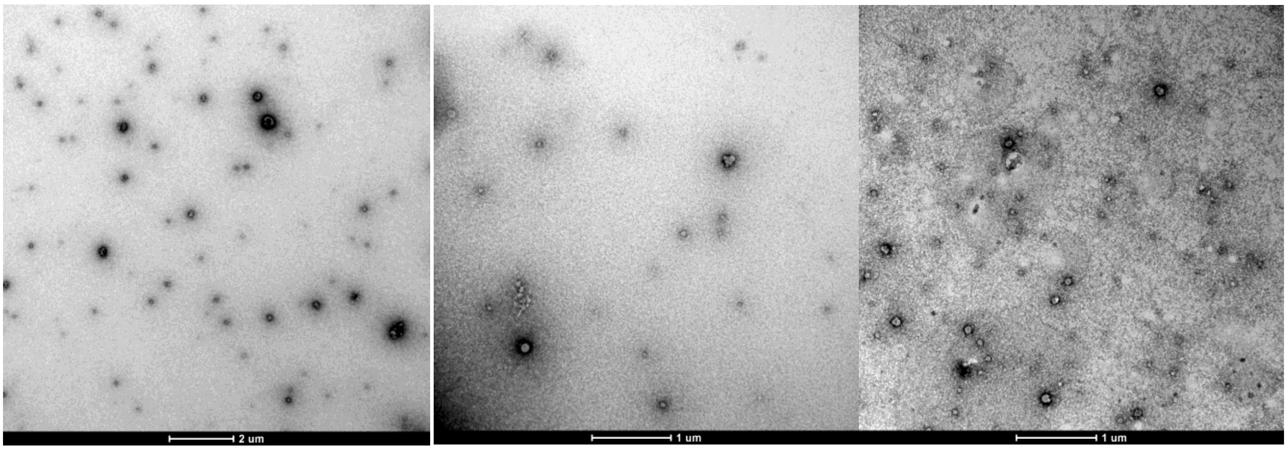

Figure S44. Additional TEM images for PEPG SUVs treated with peptide Oct-K2569-Lol at a P/L = 1/3. Comparison with pristine SUVs (panel on the left side).

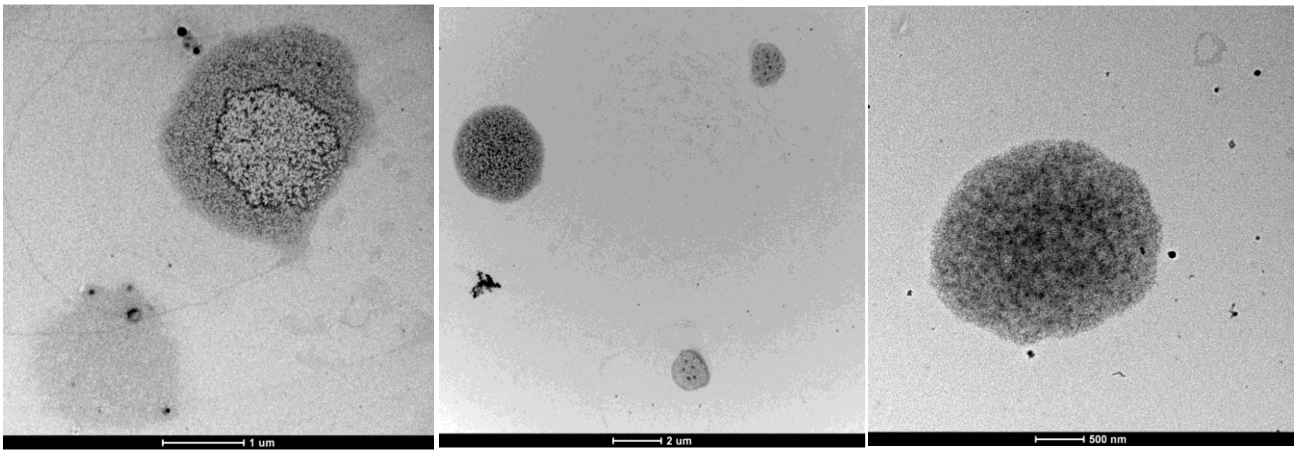

Figure S45. Additional TEM images for PEPG SUVs treated with peptide Oct-K2569-Thym at a P/L = 1/3.

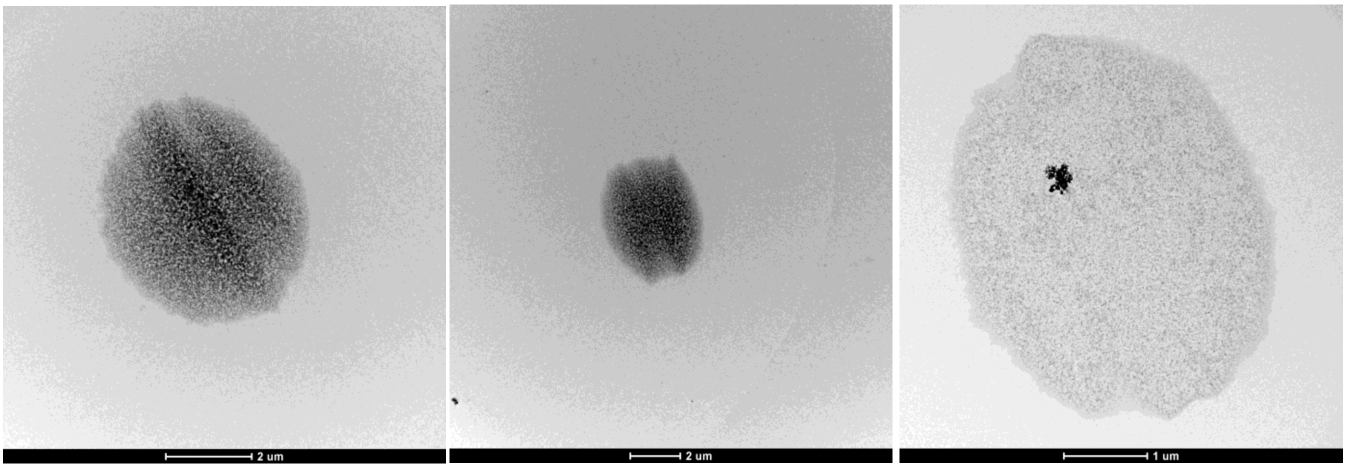

Figure S46. Additional TEM images for PEPG SUVs treated with peptide Ac-K2569-Thym at a P/L = 1/3.
